# Canonical PRC2 function is essential for mammary gland development and affects chromatin compaction in mammary organoids

**DOI:** 10.1371/journal.pbio.2004986

**Published:** 2018-08-06

**Authors:** Ewa M. Michalak, Michael J. G. Milevskiy, Rachel M. Joyce, Johanna F. Dekkers, Paul R. Jamieson, Bhupinder Pal, Caleb A. Dawson, Yifang Hu, Stuart H. Orkin, Warren S. Alexander, Geoffrey J. Lindeman, Gordon K. Smyth, Jane E. Visvader

**Affiliations:** 1 ACRF Stem Cells and Cancer Division, The Walter and Eliza Hall Institute of Medical Research, Parkville, Victoria, Australia; 2 Department of Medical Biology, The University of Melbourne, Parkville, Victoria, Australia; 3 Bioinformatics Division, The Walter and Eliza Hall Institute of Medical Research, Parkville, Victoria, Australia; 4 Department of Pediatric Oncology, Dana-Farber Cancer Institute and Division of Hematology/Oncology, Boston Children’s Hospital, Harvard Stem Cell Institute, Harvard Medical School, Boston, Massachusetts, United States of America; 5 Cancer and Haematology Division, The Walter and Eliza Hall Institute of Medical Research, Parkville, Victoria, Australia; 6 Department of Medicine, University of Melbourne, Parkville, Victoria, Australia; 7 Familial Cancer Centre, Royal Melbourne Hospital and Peter MacCallum Cancer Centre, Parkville, Victoria, Australia; 8 School of Mathematics and Statistics, The University of Melbourne, Parkville, Victoria, Australia; University of Cambridge Department of Pathology, United Kingdom of Great Britain and Northern Ireland

## Abstract

Distinct transcriptional states are maintained through organization of chromatin, resulting from the sum of numerous repressive and active histone modifications, into tightly packaged heterochromatin versus more accessible euchromatin. Polycomb repressive complex 2 (PRC2) is the main mammalian complex responsible for histone 3 lysine 27 trimethylation (H3K27me3) and is integral to chromatin organization. Using *in vitro* and *in vivo* studies, we show that deletion of *Suz12*, a core component of all PRC2 complexes, results in loss of H3K27me3 and H3K27 dimethylation (H3K27me2), completely blocks normal mammary gland development, and profoundly curtails progenitor activity in 3D organoid cultures. Through the application of mammary organoids to bypass the severe phenotype associated with *Suz12* loss *in vivo*, we have explored gene expression and chromatin structure in wild-type and *Suz12*-deleted basal-derived organoids. Analysis of organoids led to the identification of lineage-specific changes in gene expression and chromatin structure, inferring cell type–specific PRC2-mediated gene silencing of the chromatin state. These expression changes were accompanied by cell cycle arrest but not lineage infidelity. Together, these data indicate that canonical PRC2 function is essential for development of the mammary gland through the repression of alternate transcription programs and maintenance of chromatin states.

## Author summary

The formation of mammary glands requires the tight regulation of many genes that govern cell fate decisions in the cells that form them. However, most of these genes remain undefined. The Polycomb repressive complex 2 (PRC2) has a role in gene silencing, and it is comprised of several subunits, which include either Enhancer of Zeste homolog 2 (Ezh2) or Ezh1 in combination with Suppressor of Zeste 12 protein homolog (Suz12) and embryonic ectoderm development (EED). Dysregulation of these subunits can lead to breast cancer. Although previous studies have analyzed the contribution of these complexes in mammary epithelium, failure to inactivate all canonical PRC2 complexes has made this task difficult. Deletion of *Suz12* resulted in nonfunctional PRC2 and led to growth defects in mammary epithelial cells in vivo and in vitro. Here, we have used a 3D mammary organoid system to circumvent the lethality associated with *Suz12* loss and studied chromatin dynamics and gene expression of PRC2 complexes. Our results suggest that loss of all canonical PRC2 complexes results in failure to repress transcriptional programs associated with early commitment and differentiation of mammary epithelial cells.

## Introduction

A central question in biology is how different cell types maintain distinct cell fates despite containing the same genetic material. Organization of DNA into open or closed chromatin states by posttranslational modifications (PTMs) of histones has emerged as a critical mechanism underpinning cell diversity and reflecting lineage-specific gene expression, developmental programs, or disease processes [1]. The highly conserved Polycomb repressive complex 2 (PRC2), which catalyzes trimethylation of histone 3 on lysine 27 (H3K27me3), is associated with global gene repression and suppression of alternative differentiation programs. The canonical PRC2 complex is composed of the intimately associated core proteins histone methyltransferase Enhancer of Zeste homolog 2 (Ezh2) or an alternative related subunit Ezh1 [2], embryonic ectoderm development (Eed), Suppressor of Zeste 12 protein homolog (Suz12), and histone-binding protein accessory proteins [3]. Upon recruitment of PRC2 to chromatin, Ezh2/Ezh1 deposits the H3K27me3 mark associated with chromatin compaction [3]. PRC2 is required for deposition of H3K27me3 and for maintenance of this PTM upon cell division [4]. Additionally, the requirement of PRC2 activity for H3K27 mono- and dimethylation (H3K27me1 and H3K27me2) remains unclear [5]. Early studies showed that both Suz12 and Eed are nonredundant and essential for a functional PRC2 complex [6]. Deletion of *Suz12* or *Eed* resulted in elevated expression of Hox genes in *Drosophila* and mammalian cells and marked increases in gene networks that control developmental lineages [7,8] because of loss of PRC2 integrity and H3K27me3-mediated repression [9]. In contrast to Eed and Suz12, Ezh2 function can be compensated, partially [10] or completely [11], by Ezh1. Nevertheless, like mice lacking *Suz12* or *Eed*, *Ezh2*-deficient mice are not viable and die during early implantation stages [6].

Members of PRC2—in particular, Ezh2—are often found dysregulated in human cancers. In breast cancer, Ezh2 overexpression is associated with aggressive breast cancers and poor prognosis and inversely correlated with H3K27me3 expression [12]. It remains unclear whether Ezh2 overexpression is a consequence or cause of breast oncogenesis [13]. High levels of Ezh2 may not be sufficient to induce mammary tumors in mice [14], suggesting additional driver mutations are required. To further understand the basis of this dysregulation in cancer, it is imperative to determine the normal functions of PRC2 and Ezh2 in maintaining gene expression programs in the mammary gland.

The mammary gland in both humans and mice is a bilayered structure composed of two cellular lineages: an inner luminal layer and an outer myoepithelial layer that contacts the basement membrane [15]. There is increasing evidence for a differentiation hierarchy composed of stem cells, committed progenitors, and mature epithelial cells [15]. In the steady state, mouse mammary stem cell (MaSC)/basal, luminal progenitor, and mature luminal cell subsets display distinct patterns of H3K27me3 [16]. The MaSC/basal subset demonstrates the lowest levels of H3K27me3. Higher levels of H3K27me3 correlate with reduced gene expression and increase upon cell specialization [16]. These data support a model whereby mammary epithelial cell (MEC) differentiation requires narrowing of transcriptional programs and suppression of alternate cell fates. Accordingly, genetic ablation or knock-down of *Ezh2* in the mammary gland resulted in a developmental delay [16,17] but did not entirely prevent mammary gland development. This is likely due to residual H3K27 methylation, presumably established through compensatory methyltransferase activity of Ezh1. It is therefore probable that studies to date have underestimated the importance of PRC2 in directing mammary cell fate.

To reexamine the contribution of the canonical PRC2 complex to mammary gland development, we deleted *Suz12* in vivo and in mammary organoids [18]. Here, we show a nonredundant function for *Suz12* in mammary progenitor cells due to loss of PRC2 function. Similar findings were made upon deletion of *Eed*. Through the application of assay for transposase-accessible chromatin using sequencing (ATAC-seq) to probe chromatin accessibility in *Suz12*-deficient organoid cultures, we have identified regions of PRC2-dependent chromatin compaction and consequent changes in gene expression. Interestingly, the chromatin state in basal-derived organoids was reliant on PRC2 function, and *Suz12* deletion led to gene de-repression, thus highlighting a crucial role for PRC2 in the maintenance of chromatin states within the mammary epithelial hierarchy.

## Results

### *Suz12* or *Eed* deletion results in marked delay of mammary ductal outgrowth and perinatal lethality

Mammary gland development proceeds through distinct phases that include puberty and cycles of pregnancy, lactation, and involution. Suz12 expression, like Ezh2 [16,17], was detected at all stages of mammary gland development (S1A Fig) but was particularly high during puberty (4–6 week old mice). To examine the role of Suz12 in the mammary gland, we crossed mice bearing floxed *Suz12* alleles with Cre transgenic mice that express Cre under control of the mouse mammary tumor virus promoter (MMTVcre). Genotyping of offspring revealed that two-thirds of MMTVcre^T/+^Suz12^fl/f^ mice did not survive to weaning (S1 Data), and examination of newborn pups revealed abnormal lung development (S1B Fig), likely due to activity of MMTVcre in this tissue [19], and consistent with the reported role for PRC2/Ezh2 in lung [20].

**Fig 1:**
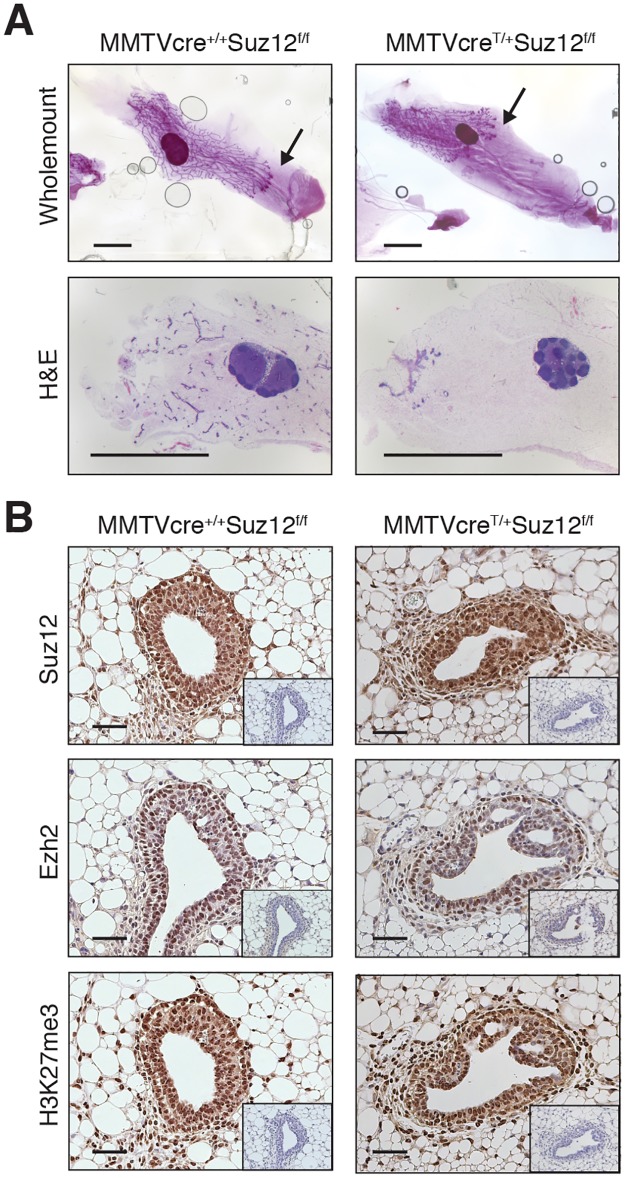
Deletion of *Suz12* selects for mammary outgrowths composed of cells that retain PRC2 function. Representative images of whole mounts, HE stained sections (A), and immunohistochemical staining for Suz12, Ezh2, and H3K27me3 (B) in mammary glands from 6 week old MMTVcre^T/+^Suz12^f/f^ and control mice. Arrows in whole mounts indicate the leading edge of the mammary epithelium. Isotype-control stained sections are shown in the inset. Scale bars: 4 mm (whole mounts, HE) and 50 μm (immunohistochemistry). Ezh2, Enhancer of Zeste homolog 2; HE, hematoxylin–eosin; H3K27me3, histone 3 lysine 27 trimethylation; PRC2, Polycomb repressive complex 2; Suz12, Suppressor of Zeste 12 protein homolog.

MMTVcre^T/+^Suz12^f/f^ mice that survived beyond birth appeared normal and did not differ from wild-type (Wt) mice with respect to bodyweight (S1C Fig). Examination of whole mounts and histological sections of mammary glands from MMTVcre^T/+^Suz12^f/f^ mice during puberty revealed a heterogeneous phenotype. Some glands were indistinguishable from Wt or heterozygous littermates, while others comprised a small ductal tree, characteristic of the rudimentary ductal tree found in newborn mice (*n* = 4) (Fig 1A and S1D Fig). These small but otherwise normal mammary glands indicate that gene deletion leads to severe impairment of ductal growth during puberty [21,22]. Notably, *Suz12* mRNA expression in MMTVcre^T/+^Suz12^f/f^ and MMTVcre^T/+^Suz12^f/+^ glands was found to be comparable (S1E Fig), demonstrating that these ductal structures were derived from epithelial cells in which *Suz12* had not been deleted. Moreover, protein expression of Ezh2, Suz12, and H3K27me3 was retained in sections from 5–6 week old MMTVcre^T/+^Suz12^f/f^ mammary glands, indicative of strong selection against *Suz12*-deleted cells and retention of a functional PRC2 complex in the mammary epithelium (Fig 1B). Cell fate was also retained as assessed by immunostaining for estrogen receptor (ER), progesterone receptor (PR), and forkhead box A1 (Foxa1) (S1F Fig).

We next determined whether deletion of the second core subunit of canonical PRC2 member *Eed* produced a similar phenotype to deletion of *Suz12*. As seen with *Suz12* deletion, conditional deletion of *Eed* with MMTVcre resulted in perinatal lethality of mice (S1 Data), and surviving mice appeared normal, except for a pronounced absence or delay in growth of the ductal tree (S2A Fig). Similar to *Suz12* deletion, *Eed* mRNA expression was found to be indistinguishable between mammary epithelium from MMTVcre^T/+^Eed^f/f^ and Wt mice (S2B Fig). In addition, Eed, Ezh2, and H3K27me3 proteins were detected by immunofluorescence (IF) and immunohistochemistry (IHC) at comparable levels to those in Wt mammary glands (S2C and S2D Fig). Taken together, these results suggest that canonical PRC2 complexes are critical for the proliferation and/or survival of MECs, and cells deleted for PRC2 function cannot contribute to the developing mammary gland.

### Suz12 is required for the integrity of PRC2 and histone H3K27me2/me3 in MECs

**Fig 2:**
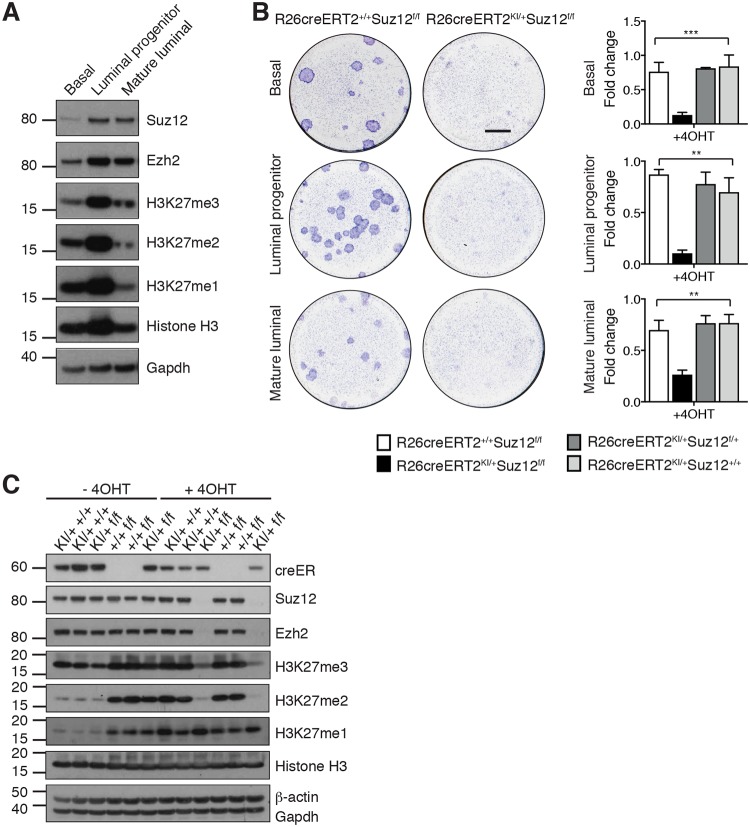
Suz12 is essential for MaSC and progenitor cell activity and integrity of the PRC2 complex. (A) Western blot analysis of PRC2 proteins and histone marks in basal/MaSC-enriched (Basal, CD29^hi^CD24^+^), committed luminal progenitor (CD29^lo^CD24^+^CD14^+^), and mature luminal cells (CD29^lo^CD24^+^CD14^−^) populations sorted from 10 week old C57BL/6 mice. Molecular mass in KDa of the protein ladder are shown on the left-hand side. (B) Representative images of Giemsa-stained colonies (left) and quantification of colonies (right) grown from sorted epithelial populations from R26creERT2^KI/+^Suz12^f/f^ mice and littermate controls in the presence or absence of 4OHT. Basal, luminal progenitor, and mature luminal cell populations (defined in A) were sorted from R26creERT2^KI/+^Suz12^f/f^ mice and control genotypes and 100 cells plated onto irradiated 3T3s. Colony formation was assessed 1 week following addition of 4OHT to induce Suz12 deletion. Mean ± S.E.M. (*n* = 3–5 independent mice per genotype performed in duplicate). ** *P* < 0.01 for KI/+ f/f compared with all other genotypes (one-way ANOVA for multiple comparisons). Scale bars: 2 mm. (C) Western blot analysis of protein expression in MECs from R26creERT2^KI/+^Suz12^f/f^ mice and the indicated control genotypes following addition of 4OHT to induce *Suz12* deletion on day 2. Cells were cultured for 1 week prior to preparation of protein lysates. Molecular mass in KDa of the protein ladder are shown on the left. Individual quantitative observations can be found in S6 Data. 4OHT, 4-hyrdoxytamoxifen; Ezh2, Enhancer of Zeste homolog 2; GAPDH, glyceraldehyde 3-phosphate dehydrogenase; H3K27me1, histone 3 lysine 27 monomethylation; H3K27me2, histone 3 lysine 27 dimethylation; H3K27me3, histone 3 lysine 27 trimethylation; MaSC, mammary stem cell; MEC, mammary epithelial cell; PRC2, Polycomb repressive complex 2; Suz12, Suppressor of Zeste 12 protein homolog.

To investigate which MEC subsets were affected by Suz12 loss, we acutely deleted *Suz12* using the inducible and conditional Rosa26-creERT2 (R26creERT2) mouse model. R26creERT2^KI/+^Suz12^f/f^ mice injected with tamoxifen die within 2 weeks because of hematopoietic failure (personal communication, S. Lee to E. Michalak), thus precluding the use of this model for in vivo studies. Basal/MaSC-enriched (basal, CD29^hi^CD24^+^), committed luminal progenitor (CD29^lo^CD24^+^CD14^+^), and mature luminal (CD29^lo^CD24^+^CD14^−^) cell subsets were sorted from R26creERT2^KI/+^Suz12^f/f^ and control mice and subjected to in vitro colony-forming assays to determine the effect of *Suz12* deletion on the activity of mammary progenitor cells following induction by 4-hydroxytamoxifen (4OHT). Consistent with expression of Suz12 in all mammary epithelial subsets (Fig 2A), we observed fewer colonies in 2D colony forming assays on irradiated feeder cells (Fig 2B) upon induction of *Suz12* deletion. In contrast to the hematopoietic system, in which loss of one allele of *Suz12* [14] or *Eed* [23] enhances the activity of stem cells, deletion of one *Suz12* allele did not affect the clonogenic activity of progenitor cells (Fig 2B). Unsorted MECs efficiently deleted *Suz12* and yielded sufficient protein for western blot analysis (Fig 2C). As expected, Ezh2 protein was completely lost upon *Suz12* deletion, while *Ezh2* mRNA levels were not reduced (S3A Fig), suggesting rapid Ezh2 protein degradation leads to the absence of functional PRC2 upon *Suz12* deletion. This is supported by the loss of H3K27me2 and H3K27me3 that accompanies *Suz12* deletion (Fig 2C). Conversely, expression of the active H3K27me1 mark [4] did not change. No change in cleaved poly ADP ribose polymerase (PARP) was detected, suggesting that *Suz12*-deleted MECs do not undergo appreciable apoptosis. However, we observed increased expression of cyclin-dependent kinase p16 and p19 alternate reading frame (p19Arf), consistent with a known role for Ezh2/PRC2 in regulating *cdkn2a* gene expression (S3B Fig). These data suggest that Suz12 exerts an essential function in the mammary gland through maintenance of functional tri- as well as dimethylation of target loci.

### *Suz12* deletion diminishes mammary organoid proliferation

**Fig 3:**
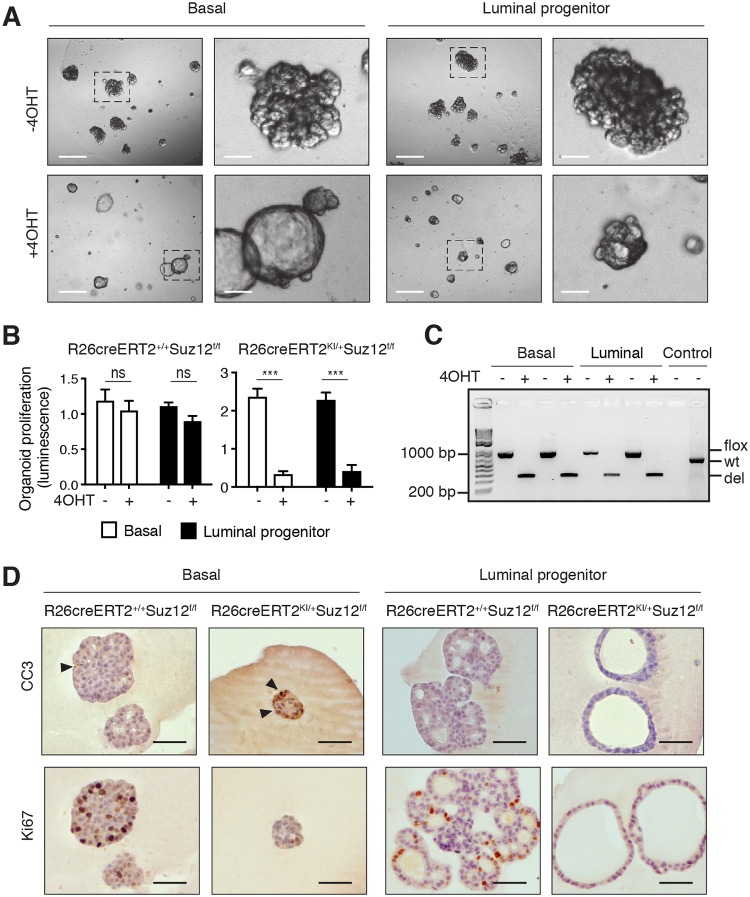
*Suz12* loss perturbs the growth of mammary organoids. (A) Representative best-focus Z stack and enlarged inset images of 2 week old organoids grown from single basal or luminal progenitor cells from R26creERT2^KI/+^Suz12^f/f^ mice. Organoids were left untreated or treated with 4OHT on day 4 of culture. Scale bars: 200 μm and 40 μm (inset). (B) Quantification of organoid proliferation in 14–16 day old cultures from R26creERT2^KI/+^Suz12^f/f^ mice or control mice described in (A), as measured by the cell titer-glow cell viability assay. Values were normalized to the average of the conditions per experiment. Mean ± S.E.M. (basal, *n* = 4–5 experiments; luminal, *n* = 6–7 experiments). *** *P* < 0.0001 (*t* test). (C) Image of genotyping PCR performed on 2 week old organoids described in (A) to distinguish the Wt, floxed (flox), and recombined (del) Suz12 alleles in basal- or luminal-derived organoids derived from 2 independent R26creERT2^KI/+^Suz12^f/f^ mice. Controls included no DNA (left) and Suz12 Wt DNA (right). The size in bp of the DNA ladder is shown on the left-hand side. (D) Immunohistochemical staining for CC3 and Ki67 on 2 week old organoids from R26creERT2^KI/+^Suz12^f/f^ mice or control mice, treated with 4OHT on day 4 of culture. Arrowheads indicate CC3-positive cells. Scale bars: 400 μm. Individual quantitative observations can be found in S6 Data. 4OHT, 4-hydroxytamoxifen; CC3, cleaved caspase 3; Suz12, Suppressor of Zeste 12 protein homolog; Wt, wild-type.

To develop a system more amenable to molecular studies, we employed a 3D mammary organoid system, in which organoids grown from single cells in defined medium recapitulate many features of mammary tissue architecture and function [18]. Single basal or luminal progenitor cells sorted from R26creERT2^KI/+^Suz12^f/f^ mice were plated, and deletion of *Suz12* was induced by addition of 4OHT on day 1. *Suz12* loss resulted in diminished numbers and smaller basal- and luminal progenitor–derived organoids over a 2 week culture period. Notably, PCR analysis of the resulting organoids revealed that selection against cre-mediated excision of both floxed alleles of *Suz12* had occurred (S3C Fig). To circumvent this issue, small organoids were allowed to form before induction of deletion. Indeed, addition of 4OHT on day 4 resulted in smaller cystic-like organoids (Fig 3A and 3B) composed of *Suz12*-deleted cells (Fig 3C and S3D and S3E Fig) that had markedly reduced levels of H3K27me3 (S3E Fig). There were no notable differences in cleaved caspase 3 (CC3) in 2 week old *Suz12*-deleted organoids, but rather, a striking decrease in proliferative (Ki67^+^) cells was evident (Fig 3D). Consistent with the idea that cells lacking Suz12 are nonproliferative, repassaging resulted in the expansion of rare cells that had escaped *Suz12* deletion (S3F and S3G Fig). Together, these data suggest that loss of Suz12 reduces the fitness and proliferation of mammary organoids.

### Suz12 loss results in changes to chromatin accessibility and gene transcription

**Fig 4:**
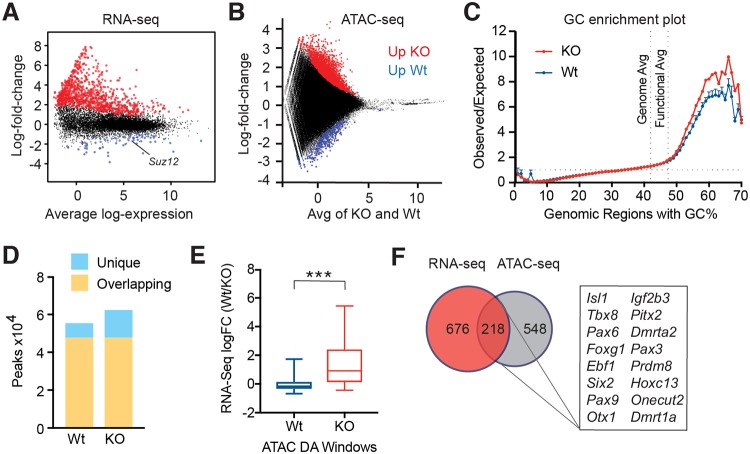
*Suz12* deletion in basal-derived organoids results in changes in chromatin accessibility and de-repression of transcriptional programs. (A) MD plot showing log2-fold expression changes versus average log2-expression identified by RNA-seq in *Suz12*-deleted basal-derived organoids compared with Wt organoids. Up- and down-regulated genes with changes significantly greater than 1.5-fold are highlighted in red and blue, respectively (Treat-FDR < 0.05). *Suz12* expression is indicated. (B) MD plot showing windows identified by 150 bp tiling as significantly DA by ATAC-seq analysis of *Suz12*-deleted basal-derived organoids, compared with Wt average log-expression. Windows that were significantly up- or down-regulated are shown in red or blue, respectively. (C) A plot showing GC nucleotide enrichment in genomic regions of ATAC-seq reads of *Suz12*-deleted basal-derived organoids, compared with Wt. Shown is the average of the genome and functional DNA elements in the mouse genome. (D) MACS Peaks calling analysis of *Suz12*-deleted basal-derived organoids (KO), compared with Wt. (E) Box and whisker plot of DA genes found to be associated with windows found in (B) by ATAC-seq analysis of Wt and *Suz12*-deleted basal-derived organoids (KO), and their corresponding expression by RNA-seq analysis. Whiskers represent the 5%–95% intervals. *** *P* < 0.0001 (unpaired *t* test, Welch’s correction). (F) Venn diagram showing the overlap in up-regulated DE genes identified by RNA-seq and DA genes by ATAC-seq in *Suz12*-deleted basal-derived organoids. Individual quantitative observations can be found in S6 Data. ATAC-seq, assay for transposase-accessible chromatin using sequencing; DA, differentially accessible; DE, differentially expressed; FDR, false discovery rate; KO, knockout; MACS, model-based analysis of ChIP-seq; MD, mean-difference; RNA-seq, RNA-sequencing; Wt, wild-type.

To determine potential target genes that are dependent on Suz12 and therefore canonical PRC2 function, we performed RNA sequencing (RNA-seq) analysis of *Suz12* Wt or *Suz12*-deleted basal- and luminal-derived mammary organoids grown from R26creERT2^KI/+^Suz12^f/f^ mice. *Suz12* was significantly down-regulated in both populations (Fig 4A, S4A Fig and S2 Data), and deletion of exon 5 was confirmed at the targeted locus (S4B Fig). In both basal- and luminal-derived organoids, the majority (>86%) of differentially expressed (DE) genes were up-regulated rather than down-regulated upon *Suz12* deletion, consistent with the repressive role of PRC2 (Fig 4A, S4A and S4C Fig). A large overlap was apparent between up- and down-regulated DE genes (S4C Fig), as well as a strong correlation with gene expression changes (S4D Fig) in *Suz12*-deleted basal- versus luminal-derived organoids. This is consistent with a global effect of *Suz12* deletion on PRC2 stability in MECs. Gene ontology (GO) enrichment analysis found that common DE down-regulated genes were significantly enriched for metabolic processes and H3K27 methylation, while common DE up-regulated genes were enriched for development and morphogenesis GO terms (S4C Fig).

To explore whether the changes in gene expression accompanying loss of *Suz12* reflect alterations in chromatin compaction associated with loss of repressive H3K27me2/me3 marks, we performed global mapping of chromatin accessibility using ATAC-seq. Differential accessibility analysis identified 2,767 windows, of which 2,415 (87.3%) were more accessible upon *Suz12* deletion (Fig 4B and S3 Data). Further, 1,377 of these windows overlapped with the transcription start site or gene body of 766 independent genes in *Suz12*-deleted basal organoids (referred to as differentially accessible [DA] genes from hereon). Nevertheless, the frequency of reads mapping to nucleosome-free regions versus mononucleosomes was not significantly altered. We observed only minor changes to insert length, suggesting no gross changes in nucleosome occupancy (S5A and S5B Fig), and a comparison of GC enrichment indicated a minor increased frequency of highly GC-rich regions, usually found at regulatory elements within promoters (Fig 4C). Moreover, model-based analysis of ChIP-seq (MACS) peaks analysis showed a small number of unique peaks upon *Suz12* deletion (Fig 4D). Next, we compared the DA windows identified by ATAC-seq with gene expression from their associated gene to determine if changes in chromatin compaction elicited by *Suz12* deletion were sufficient to induce changes in gene transcription. A strong correlation was evident between open chromatin and transcriptional up-regulation in *Suz12*-deleted basal-derived organoids (Fig 4E). However, analysis of Wt organoids did not reveal a correlation between genes assigned from ATAC-seq analysis and changes in their expression. Almost one-third (28%) of genes that became more accessible upon *Suz12* deletion were significantly up-regulated by RNA-seq (Fig 4F). These 218 common genes included *Cdkn2a* (S6A Fig and S4 Data), a known target of Ezh2-mediated repression that encodes p16 and p19Arf, and transcription factors (TFs) involved in diverse lineage commitment, including *Foxd1* and homeobox TFs (S6B and S6C Fig), and accordingly, represented sequence-specific DNA-binding GO terms (S5 Data). Notably, confocal imaging of organoids confirmed the persistence of basal and luminal cells in *Suz12*-deleted organoids (S6D Fig), suggesting lineage fidelity was maintained.

A similar ATAC-seq analysis was applied to *Suz12*-deleted luminal-derived organoids (S7A and S7B Fig), which revealed an increase in reads associated with nucleosomes (S5A and S5B Fig). These longer fragments were enriched in promoter-flanking and transcribed regions [24] consistent with increased expression of DE genes. Despite detecting many unique DA peaks (S7C Fig), particularly associated with promoters and intragenic/gene bodies (S7D Fig), the corresponding windows were less strongly associated with DE genes found by RNA-seq (S7E and S7F Fig).

Comparison of the *Suz12*-deleted gene expression signatures derived for both basal and luminal organoids showed that they were most similar to claudin-low and normal-like breast tumors (S7G Fig), similar to that observed for the basal/MaSC cell signature [25]. Likewise, the gene expression profile of *Ezh2*-deleted MECs was most concordant with claudin-low breast tumors [16]. The striking similarity between gene expression signatures of organoids lacking key PRC2 complex genes and claudin-low breast tumors—which exhibit a metaplastic, clinically aggressive phenotype in patients—suggests that PRC2 may play a role in tumor phenotype and behavior.

## Discussion

Using in vivo and in vitro studies, we show that deletion of a core component (either Suz12 or Eed) of the canonical PRC2 complexes leads to a complete block in mammary gland development in vivo and markedly curtails progenitor cell activity in vitro. Strikingly, *Suz12-* or *Eed*-deleted cells were strongly selected against and could not contribute to the developing mammary gland. This resulted in very small glands consisting of Wt cells. These findings are consistent with previous reports for nonredundant genes that are required for stem cell function [26]. Assays using primary MECs and organoids suggest that this is due in part to de-repression of *cdkn2a*, resulting in increased p16 and p19 expression and reduced proliferation owing to cell cycle arrest. Thus, previous studies [16,17] have underestimated the importance of PRC2 in governing mammary progenitor activity and differentiation, owing to some functional redundancy between Ezh1 and Ezh2. Moreover, these results are consistent with PRC2 promoting mammary epithelial expansion, rather than inhibiting it [27]. By exploiting mammary organoids to bypass the severe developmental phenotype associated with loss of Suz12, we examined changes in chromatin structure in the presence and absence of PRC2 and correlated these with gene expression changes.

PRC2 complexes are reported to colocalize with H3K27me3 on the promoters of around 10% of all genes [5]. Upon *Suz12* deletion, we observed comparable changes in gene expression (7%–11%) in both basal- and luminal-derived organoids. There was significant overlap between the up-regulated genes in basal- and luminal-derived organoids, and the magnitude of change was highly correlated. Assessment of chromatin accessibility with ATAC-seq indicated that the chromatin of basal-derived organoids is more open upon Suz12 loss. Recent findings in human breast epithelial subsets [28] predict that bivalent promoters and primed enhancers would be the most affected by loss of H3K27me3 in *Suz12*-deleted basal-derived organoids. Since we did not observe a significant increase in GC content of DA reads identified by ATAC-seq, the mechanism is most likely to involve enhancers. Coupling RNA-seq with ATAC-seq analysis in basal-derived organoids revealed that deletion of *Suz12* led to more than one quarter of genes becoming significantly transcriptionally active coincident with more accessible chromatin. We have previously shown that the MaSC/basal subset demonstrates the lowest levels of H3K27me3 across transcriptional start sites (TSSs) [16] while also producing slightly less RNA overall [29]. This observation supports the notion that this gene subset marked by H3K27me3 is transcriptionally regulated through H3K27me3-mediated repression accompanied by chromatin compaction and is dependent on canonical PRC2 function. Our results indicate that open chromatin is a good predictor of gene activation in basal-derived organoids. In the luminal compartment, there is an increased level of H3K27me3 [16], while these cells are more transcriptionally active overall [29]. In luminal-derived organoids, loss of PRC2 resulted in increased chromatin accessibility and an increase in reads mapping to regions with high GC content. In contrast to basal-derived organoids, the low correlation with gene expression in luminal-derived organoids suggests that additional mechanisms beyond chromatin accessibility serve to ensure proper gene repression. This may be due to DNA methylation or the combination of additional activating and repressive histone marks that maintain gene repression in this more committed cell type, as observed for human luminal cells [28].

Our studies indicate that PRC2 is responsible for both H3K27me3 and H3K27me2 marks in MECs, as evidenced by their loss in *Suz12*-deleted cells. Notably, we show here that the absence of these repressive marks through deletion of a single protein results in large changes in gene expression, consistent with the notion that chromatin states are the sum of a limited repertoire of PTM combinations on histone tails. While these data do not exclude noncanonical functions in the presence of a functional PRC2 complex [30], they suggest that Suz12 is a limiting factor for expression of Ezh2 protein and thus a functional PRC2 complex in the mammary epithelium.

In the context of breast cancer, our data indicate that *Suz12*-deleted organoids, like *Ezh2*-null basal cells [16], displayed similarities with signatures of claudin-low breast cancers, which are thought to arise from MaSCs. While Ezh2 up-regulation was initially associated with aggressive breast cancers, several studies now indicate that Ezh2 overexpression may be a consequence rather than a cause of breast cancer [13]. Indeed, deletion of Ezh2 accelerated tumors in a mouse model of *Brca1*-deleted breast cancer [31] and in a breast cancer model of Notch activation [13]. Additionally, the status of the *cdkn2a* locus of an individual tumor might predict its response to Ezh2 loss. The observation that Suz12 deficiency in normal mammary cells leads to de-repression of *cdkn2a*, paralleling that seen with loss of Ezh2 [32], would predict that loss of PRC2 function in breast cancer would result in decreased tumor proliferation via up-regulation of p16^Ink4a^ and p14Arf expression. However, if this locus is perturbed, as is seen in many breast cancers [33], then loss of Ezh2 might have quite different consequences. Further work will be required to determine if loss rather than overexpression of PRC2 contributes to tumor progression in other cellular contexts.

In summary, our findings establish an essential role for PRC2 in the maintenance of mammary progenitor function and point to a critical function for PRC2 in maintaining chromatin states to ensure appropriate gene expression in MECs.

## Methods

### Ethics statement

All animal experiments were conducted using mice bred at and maintained in our animal facility according to the Walter and Eliza Hall Institute of Medical Research Animal Ethics Committee guidelines, approval number 2017.002.

### Mice

Suz12^f/f^ [34], Eed^f/f^ [35], Ezh2^f/f^ [36], MMTV-cre (line A) [37], and R26creERT2 [38] gene-targeted mice have been described previously. CD4cre-Eed^f/f^ T lymphocytes [39] were a gift from R. Allan. MMTV-cre mice were maintained as a pure strain on an FVB/N background, R26creERT2 mice were on a C57BL/6 background, and Ezh2, Eed, and Suz12 conditional knockout mice were on either a C57BL/6 or mixed FVB/N and C57BL/6 background. For analysis of postnatal day 1 lungs, adult female mice were subjected to timed pregnancies and injected with 0.2 mg progesterone on days 17 and 18 of pregnancy to delay parturition. At 19.5 days of pregnancy, pups were recovered by caesarian section and monitored for breathing for 1 hour with constant stimulation before collection of lungs into Bouin’s solution.

### Mammary cell preparation and cell sorting

Primary mouse MEC cultures were isolated from both inguinal and/or thoracic mammary glands and prepared as described [17]. MEC suspensions and flow cytometry were performed as previously described [40]. Antibodies against mouse antigens were purchased from Biolegend and included CD24-PB (#101820), CD31 (#102410), CD45 (#103112), and Ter119 (#116212) conjugated to APC, CD29–FITC (#102206), and CD14-PE (#123309). Cells were sorted on a FACSAria or FACSDiva (BD PharMingen) and manually counted prior to plating on irradiated fibroblast feeders as described [40]. After 7 days, colonies were fixed and stained with Giemsa and manually counted. To induce R26creERT2-mediated deletion, 0.1 μM 4OHT was added to culture medium for 20 hours on day 1 of culture.

### Organoid culture

Organoids were cultured as described previously [18] in 8 μl BME (Cultrex) drops (70 basal and 60 luminal progenitor cells per drop) on nontreated 24 well plates and covered by Advanced DMEM/F12 supplemented with growth factors excluding Wtn3a or FGF2. Rock inhibitor (Y-27632) was added to culture medium for the first 4 days, and the medium was refreshed every 2–3 days. To induce R26creERT2-mediated deletion, 0.1 μM 4OHT was added to culture medium for 16–20 hours on day 1 or day 4 of culture. Organoids were photographed using best focus projection images on the Nikon TiE System software after 12–14 days in culture. Prior to proliferation measurements, RNA-seq, or ATAC-seq analysis, 12–16 day old organoids were dissociated into single cells using TrypLE express (Thermo Fisher Scientific). Cell suspensions were mixed 1:1 with CellTiter-Glo Luminescent Cell Viability Assay (Promega) prior to detection of luminescence. Genomic DNA was extracted from organoids using DNeasy Blood and Tissue kit (Qiagen) and PCR used to distinguish the Wt, floxed, and recombined (deleted, referred to as “del”) Suz12 alleles as described [34].

### Histology and whole mounting

Mice were injected with BrdU Cell Labeling Reagent (0.5 mg/10 g body weight, Amersham Biosciences) 1.5 hours prior to collection. For histology, tissues were fixed in 4% paraformaldehyde overnight and embedded in paraffin. Sections (5 μm) were stained with hematoxylin–eosin (HE). For whole-mount analysis, mammary glands were harvested and fixed in Carnoy’s solution (6:3:1 of 100% ethanol, chloroform, and glacial acetic acid) and stained with Carmine alum. The extent of ductal outgrowth was measured on inguinal whole mounts as the distance from the center of the lymph node to the leading edge of the ductal mass.

### Immunostaining

IHC and IF were performed as described [16]. Paraffin-embedded sections (5 μm) were dewaxed in xylene and rehydrated through an alcohol series, blocked with 3% hydrogen peroxide, and subjected to antigen retrieval by boiling in 10 mM citrate buffer pH 6.0 for 30 seconds using a DAKO pressure cooker. Immunostaining was performed using the streptavidin-biotin peroxidase detection system as per the manufacturer’s instructions (ABC reagent, Vector Laboratories) and 3,3-diaminobenzidine was used as substrate (DAKO). In all cases, an isotype-matched control IgG was used as a negative control. The following antibodies were used: anti-BrdU (Bio Rad OBT0030), anti-Ezh2 (BD Biosciences #612667), anti-ERα (Santa Cruz sc-543), anti-PR (Santa Cruz sc-538), anti-Foxa1 (Abcam Ab23738), anti-H3K27me3 (Millipore #07–449), anti-Suz12 (Diagenode pAB-029-050), anti-Eed (R&D Systems AF5827-SP), anti-CC3 (Cell Signaling #9664L), and anti-Ki67 (Cell Signaling #12202S). Secondary antibodies were biotin-conjugated anti-rabbit IgG, anti-rat IgG and anti-mouse IgG (Vector Laboratories), anti-mouse alexa-488 (Invitrogen), and anti-rabbit alexa-647 (Invitrogen), and DAPI was used to detect nuclei (Thermo-Fischer Scientific). As described in [18], 3D imaging of organoids was performed on a SP8 Confocal microscope after staining with anti-cytokeratin 14 (Thermo-Fisher Scientific) and anti-cytokeratin 8/18 (TROMA-I, DSHB) antibodies.

### Western blot analysis

Lysates were prepared in RIPA buffer [16], and western blotting was performed as described [17]. The following antibodies were used for western blot analysis: anti-Ezh2 (BD Biosciences #612666), anti-Suz12 (Cell Signaling 3737S), anti-Eed (Millipore 05–1320), anti-H3K27me1 (Millipore 07–448), anti-H3K27me2 (Millipore 07–452), anti-H3K27me3 (Millipore 07–449), anti-Histone 3 (Millipore 07–690), anti-β-actin (Sigma A5441), anti-Gapdh (Sigma G8795), anti-ERα (Millipore 07–690), anti-Ezh1 (Millipore 07–690), anti-PARP (Cell Signaling #9542), anti-p16 (Santa Cruz M-156), and anti-p19 (Rockland Immunochemicals #200-501-891). Secondary antibodies included HRP-conjugated anti-rabbit and anti-mouse (Southern Biotech), both 1:10,000.

### ATAC-seq analysis

ATAC-seq was performed as described [24] with the following adaptations. Organoid cultures were dissociated, and 50,000 single cells were lysed and nuclei collected at 500 g for 10 minutes in lysis buffer containing 0.1% NP-40. Pelleted nuclei were tagmented with Nextera Tn5 Transposase (TDE1, Illumina FC-121-1030) for 20 minutes at 22 °C and 30 minutes at 37 °C. Transposed DNA was purified using Qiagen MinElute kit (28204) and fragments PCR amplified as described [24]. ATAC libraries were sequenced on a NextSeq using a 150H kit with 75 bp paired-end reads, and total reads were collated from 2 runs to obtain approximately 60 × 10^6^ reads per sample. Sequencing runs were pooled for each replicate and sample, and adapters were trimmed with Trim Galore! (http://www.bioinformatics.babraham.ac.uk/projects/trim_galore/) and mapped to the mm10 mouse genome using bowtie2 [41], allowing for fragments <2,000 bases in length. Mitochondrial and duplicate reads were removed using Picard-Tools (http://broadinstitute.github.io/picard/), and bam files were run through macs2 (doi: 10.1186/gb-2008-9-9-r137) for peak calling using these parameters: “—nomodel—shift—75—extsize 150—qvalue 0.05.” For DA analysis, bam files were loaded in SeqMonk (v1.37.1, https://www.bioinformatics.babraham.ac.uk/projects/seqmonk/), and probes were created using a tiling approach with 150 bp windows end to end across the genome. Raw counts were then processed through the differential gene expression pipeline within edgeR [42], and DA regions were called with an exact test and FDR < 0.05. Reads were quantified by reads per million and log2 transformed for visualizing graphically. For each gene, the 5 Kb region upstream of intron 1 was used in combination with the gene body to define the TSSs plus gene body. GC enrichment was quantified on mapped bam files using DeepTools [43] computeGCBias with an effective genome size of 2,150,570,000. These data have been deposited in the Gene Expression Omnibus (accession code GSE116431).

### RNA-seq analysis

Total RNA was extracted from basal- or luminal-derived organoids grown from single sorted luminal or basal populations from the mammary glands of R26creERT2/Suz12^f/f^ female mice using the RNeasy Mini kit (Qiagen). Two biological replicates were prepared of the basal-derived organoids and 3 biological replicates of luminal-derived. RNA-seq was carried out on an Illumina Nextseq 500 to produce 20–65 million 80 bp reads per sample. Read pairs were mapped to the mouse genome (mm10) using the subread aligner [44] implemented in the Rsubread software package. Read counts for Entrez Genes were obtained using featureCounts [45] and its inbuilt mm10 annotation. Gene information was downloaded from the NCBI on 1 February 2017. Statistical analysis used the limma [46] and edgeR [42] software packages. Genes with at least 0.5 read counts per million (cpm) in at least 2 samples were considered to be expressed and were kept in the analysis. Immunoglobulin receptor segments, ribosomal genes, predicted and pseudogenes, and obsolete Entrez IDs were filtered out. Trimmed mean of M-values (TMM) scale normalization [47] was applied, and read counts were transformed to log2-cpm with a prior count of 3. Linear models were used to test for expression differences between 4OHT treated versus untreated samples from luminal cells and from basal cells. Each organoid sample was treated as a random block, allowing for correlation between repeats [48]. Differential expression was assessed using the Treat method [49], computing empirical Bayes moderated *t* statistics relative to a fold change threshold of 1.5, and allowing for an abundance trend in the standard errors and for robust estimation of the Bayesian hyperparameters [50]. The Benjamini and Hochberg method was used to control the FDR. These data have been deposited in the Gene Expression Omnibus (accession code GSE116431).

### Expression profiles for breast cancer subtypes

Microarray expression profiles of breast tumors were downloaded from Gene Expression Omnibus series GSE18229 [51]. Probe intensities were normexp background corrected with offset 50 [52] and loess normalized using the limma package. Mouse Entrez Gene IDs were mapped to human using HUGO Gene Nomenclature Committee orthology predictions downloaded November 2016. Suz12-deficient expression signatures were computed for each tumor using a previously described method [25]. Briefly, the sum of products of RNA-seq log2-fold-changes with microarray log2-normalized intensities was computed for all genes DE in the Suz12 deficient basal- or luminal-derived organoids.

### Quantitative reverse-transcriptase PCR (qRT-PCR) analysis

For qRT-PCR analysis, total RNA from MECs was reverse-transcribed using Superscript III (Invitrogen), and cDNA were analyzed on a LightCycler 480 (Roche). Input cDNA concentrations were normalized to GAPDH. Product accumulation was evaluated using the comparative Ct method (2^−ΔΔCT^). Primer sequences were: *Ezh2* For: 5′-ACATCCCTTCCATGCAACACC-3′; *Ezh2* Rev: 5′-TCCCTCCAGATGCTGGTAACA-3′; *Eed* For: 5′-GTGAACAGCCTCAAGGAAGAT-3′; *Eed* Rev: 5′- ATAAGGTTACTCTGTGCTTC-3′; *Gapdh* For: 5′-TGACATCAAGAAGGTGGTGAAG; *Gapdh* Rev: AAGGTGGAAGAGTGGGAGTTGC-3′.

## Supporting information

S1 Fig (A) Western blot for Ezh2 and Suz12 in whole mammary gland lysates prepared at the indicated developmental timepoints. Gapdh serves as a loading control. Molecular mass in KDa of the protein ladder is shown on the left-hand side. (B) Representative images of HE-stained sections of lungs from MMTVcre^T/+^Suz12^f/f^ and control littermates recovered by caesarian section at E19.5. Examination of HE-stained sections revealed that while lungs appear to be normal in size (top), at higher magnification (bottom), lungs from MMTVcre^T/+^Suz12^f/f^ mice have fewer septa and increased mesenchyme, consistent with abnormal lung differentiation. Scale bars: 1.5 mm (top) and 50 μm (bottom). (C) Weight of 5–6 week old MMTVcre^T/+^Suz12^f/f^ mice and control littermates of the indicated genotypes. (D) Ductal extension of mammary glands from 5–6 week old MMTVcre^T/+^Suz12^f/f^ mice and control littermates of the indicated genotypes. Ductal extension is calculated as the distance from the center of the lymph node to the leading edge of the mammary outgrowth in R4 mammary gland whole mounts. Individual data points and the mean are shown. ** *P* < 0.01 for T/+ f/f compared with all other genotypes (one-way ANOVA for multiple comparisons). (D) qRT-PCR analysis of *Suz12* mRNA expression in mammary glands from 6 week old MMTVcre^T/+^Suz12^f/+^ and MMTVcre^T/+^Suz12^f/f^ mice. Expression was calculated relative to *Gapdh*, and values were normalized to the average of the conditions per experiment. Mean ± S.E.M. (*n* = 4). R26creERT2^KI/+^Suz12^f/f^ MECs treated without (Wt) or with 4OHT (ko) to delete *Suz12* were used as controls (*n* = 1). One of two experiments with two independent sets of primer pairs for *Suz12* is shown. (E) Representative images of immunohistochemical staining of terminal end buds in mammary glands from 6 week-old MMTVcre^T/+^Suz12^f/f^ and control littermates. Markers of proliferation (BrdU) and differentiation of MECs into hormone receptor positive mammary subsets (Foxa1, ER, PR) were included. Isotype-control stained sections are shown in the inset. Scale bars: 50 μm. Individual quantitative observations can be found in S6 Data. 4OHT, 4-hydroxytamoxifen; BrdU, bromodeoxyuridine; dI, days involuting; dL, days lactating; dP, days pregnant; E, embryonic day; ER, estrogen receptor; Ezh2, Enhancer of Zeste homolog 2; Foxa1, forkhead box 1A; Gapdh, glyceraldehyde 3-phosphate dehydrogenase; HE, hematoxylin–eosin; ko, knockout; MEC, mammary epithelial cell; qRT-PCR, quantitative reverse-transcriptase PCR; PR, progesterone receptor; Suz12, Suppressor of Zeste 12 protein homolog; V, virgin; Wt, wild type.
(TIF)

S2 Fig (A) Representative images of whole mounts (left) and ductal extension (right) of mammary glands from MMTVcre^T/+^Eed^f/f^ mice and control littermates of the indicated genotypes. Arrows indicate the leading edge of the mammary epithelium. Scale bars: 4 mm. Ductal extension was calculated as described in S1 Fig. Individual data points and the mean are shown. * *P* < 0.05 for T/+ f/f compared with all other genotypes (one-way ANOVA for multiple comparisons). (B) qRT-PCR analysis of *Eed* mRNA expression in mammary glands from 6–7 week old MMTVcre^+/+^Eed^f/f^ and MMTVcre^T/+^Eed^f/f^ mice. Expression was calculated relative to *Gapdh*, and values were normalized to the average of the conditions per experiment. Mean and individual values are shown (*n* = 2). CD4cre^+/+^Eed^f/f^ (f/f +/+) and CD4cre^T/+^Eed^f/f^ (f/f T/+) T lymphocytes were used as controls (*n* = 1). One of two experiments with two independent sets of primer pairs for *Eed* is shown. (C) Immunofluorescent staining for Eed in mammary glands from 6 week old MMTVcre^T/+^Eed^f/f^ and control littermates. Scale bars: 50 μm. (D) Immunohistochemical staining for Ezh2 and H3K27me3 in mammary glands from 6 week old MMTVcre^T/+^Eed^f/f^ and control littermates. Isotype-control stained sections are shown in the inset. Scale bars: 50 μm. Individual quantitative observations can be found in S6 Data. Eed, embryonic ectoderm development; H3K27me3; histone 3 lysine 27 trimethylation; qRT-PCR, quantitative reverse-transcriptase PCR.
(TIF)

S3 Fig (A) qRT-PCR analysis of *Ezh2* mRNA expression in MEC from R26creERT2^KI/+^Suz12^f/f^ mice and the indicated control genotypes following addition of 4OHT to induce *Suz12* deletion on day 2. Cells were cultured for 1 week prior to preparation of RNA. Copies of *Ezh2* are expressed relative to GAPDH. (B) Western blot analysis of protein expression in MECs from R26creERT2^KI/+^Suz12^f/f^ mice and the indicated control genotypes following addition of 4OHT to induce *Suz12* deletion on day 2. Cells were cultured for 1 week prior to preparation of protein lysates. Molecular mass in KDa of the protein ladder are shown on the left. (C) Image of genotyping PCR performed on organoids grown for 2 weeks from single basal or luminal progenitor cells from R26creERT2^KI/+^Suz12^f/f^ mice or Wt mice. Organoids were left untreated (-) or treated with 4OHT on day 1 (1) or day 4 (4) of culture. The size of Suz12 Wt, floxed (flox), and recombined (del) alleles are indicated. The size (bp) of the DNA ladder is shown on the left-hand side. (D) Immunohistochemical staining for Suz12 on 2 week old organoids from R26creERT2^KI/+^Suz12^f/f^ or control mice, treated with 4OHT on day 4 of culture. Control stained sections are shown in the inset. Scale bars: 400 μm. (E) Western blot analysis of 2 week old organoids from R26creERT2^KI/+^Suz12^f/f^ mice or control mice, treated with 4OHT on day 4 of culture. Molecular mass in KDa of the protein ladder is shown on the left-hand side. (F) Representative images of repassaged organoids grown for 2 weeks from single basal cells from R26creERT2^KI/+^Suz12^f/f^ mice, on day 1 and day 11 after passaging. Black arrowheads indicate clumps of cells that became cystic overnight after passaging. White arrowheads represent new noncystic colonies that formed from single cells. Scale bars: 200 μm. (G) Image of genotyping PCR performed on primary or repassaged organoids described in (B) after 11 days in culture. The size of Suz12 Wt, flox, and del alleles are indicated. Basal- or luminal-derived organoids were derived from 2 independent R26creERT2^KI/+^Suz12^f/f^ mice. Controls included Suz12 Wt DNA (left) and no DNA (right). The size (bp) of the DNA ladder is shown on the left-hand side. Individual quantitative observations can be found in S6 Data. 4OHT, 4-hydroxytamoxifen; GAPDH, glyceraldehyde 3-phosphate dehydrogenase; MEC, mammary epithelial cell; qRT-PCR, quantitative reverse-transcriptase PCR; Suz12, Suppressor of Zeste 12 protein homolog; Wt, wild-type.
(TIF)

S4 Fig (A) MD plot showing log2-fold expression changes versus average log2-expression by RNA-sequencing in luminal-derived organoids deleted for Suz12. Up- and down-regulated genes with changes significantly greater than 1.5-fold are highlighted in red and blue, respectively (Treat-FDR < 0.05). *Suz12* expression is indicated. (B) IGV profile of RNA-sequencing reads for *Suz12* in basal- or luminal-derived organoids left untreated or treated with 4OHT on day 4 of culture. The metrics are shown on the left side of each plot for each sample. The direction of transcription is marked by an arrow. Exon 5, around which loxP sites are situated in the Suz12 floxed allele, is indicated by the boxed region. Scale bar: 5 Kb. (C) Venn diagrams showing the number of DE down-regulated (left) and up-regulated (right) genes in *Suz12*-deleted basal- and luminal-derived organoids. Overlap in DE genes is indicated, as well as the 5 top GO terms for that gene set. (D) Scatterplot relating log2-fold gene expression changes in Suz12-deleted basal- and luminal-derived organoids. Changes in basal-derived (x-axis) and luminal-derived (y-axis) organoids are highly correlated (*P* < 1e-16). Individual quantitative observations can be found in S6 Data. 4OHT, 4-hydroxytamoxifen; DE, differentially expressed; FDR, false discovery rate; GO, Gene ontology; IGV, Integrative Genomics Viewer; MD, mean-difference; Suz12, Suppressor of Zeste 12 protein homolog.
(TIF)

S5 Fig (A) The averaged frequency of read insert length obtained by ATAC-seq for Wt and *Suz12*-deleted (ko) basal- and luminal-derived organoids relative to distance from the TSS is shown. The 40 and 178 nt peaks correspond to nucleosome-free regions and the mononucleosome, respectively. (B) An image of the ATAC-seq libraries for Wt (-) or *Suz12*-deleted (+) basal- and luminal-derived organoids, resolved on Tape-station. The ladder is in the first lane on the left-hand side. Individual quantitative observations can be found in S6 Data. ATAC-seq, assay for transposase-accessible chromatin using sequencing; ko, knockout; nt, nucleotide; TSS, transcriptional start site; Wt, wild-type.
(TIF)

S6 Fig The RNA-seq and ATAC-seq profiles around *Cdkn2a* (A), *Foxd1* (B), and *Hoxc* locus (C) are shown. The metrics are shown on the left side of each plot for each sample. The direction of transcription is marked by an arrow. Scale bars are indicated. (D) A whole-mount 3D confocal image (left) and an optical section (right) of a Wt and *Suz12*-deleted (KO) basal-derived organoid labeled for K14 and K8/18. Scale bars: 30 μm (whole mount) and 15 μm (section). ATAC-seq, assay for transposase-accessible chromatin using sequencing; RNA-seq, RNA sequencing; Wt, wild-type.
(TIF)

S7 Fig (A) MD plot showing 150 bp windows identified as significantly DA (log-fold change) by ATAC-seq analysis of *Suz12*-deleted (KO) luminal-derived organoids deleted for Suz12, compared with Wt average log-expression. Regions that were significantly up- or down-regulated are shown in red or blue, respectively. (B) A plot showing GC nucleotide enrichment in genomic regions of ATAC-Seq reads of *Suz12*-deleted (KO) luminal-derived organoids, compared with Wt. Shown are the genomic and functional DNA element average for the mouse genome. (C) MACS Peaks calling analysis of *Suz12*-deleted (KO) luminal-derived organoids, compared with Wt. (D) Analysis of genomic regions associated with significantly DA windows identified by ATAC-seq analysis of Wt and *Suz12*-deleted (KO) basal- or luminal-derived organoids. (E) Box and whisker plot of significantly DA genes found to be associated with windows found in (A) by ATAC-seq analysis of Wt and *Suz12*-deleted (KO) luminal-derived organoids, and their corresponding expression by RNA-seq analysis. Whiskers represent the 5%–95% intervals. ** *P* < 0.0028 (unpaired *t* test, Welch’s correction) (F) Venn diagram showing the overlap in up-regulated DE genes between RNA-seq and DA genes by ATAC-seq in *Suz12*-deleted luminal-derived organoids. (G) *Suz12*-deleted transcriptional signature by breast cancer tumor subtype (Claudin-low, Normal-like, Luminal A, Luminal B, Her2-positive, Basal-like). Box plots show the aggregate gene expression score in each tumor subtype [51] for genes associated with *Suz12*-deficiency in basal-derived organoids (left) and luminal-derived organoids (right). The *Suz12*-deficient expression score is highest in the claudin-low subtype and lowest in the basal and Her2 subtypes (*P* = 3.8e-10 for basal-derived and i = 6.2e-11 for luminal-derived organoids by Tukey HSD). ATAC-seq, assay for transposase-accessible chromatin using sequencing; DA, differentially accessible; DE, differentially expressed; Her2, human epidermal growth factor receptor 2; KO, knockout; MACS, model-based analysis of ChIP-seq; MD, mean-difference; RNA-seq, RNA sequencing; Suz12, Suppressor of Zeste 12 protein homolog; Wt, wild type.
(TIF)

S1 Data Genotyping results of litters from MMTVcre-Suz12^f/f^ and MMTVcre-Eed^f/f^ matings using the indicated mating pairs. Observed numbers and percentages of males, females, and total offspring of the indicated genotypes at weaning, as well as the expected mendelian frequency (expressed as percentage of total), are shown.
(XLSX)

S2 Data Significantly altered genes found by RNA-seq in *Suz12*-deleted basal- and luminal-derived mammary organoids. Length = gene length in bases (total of all exons). Remaining columns give the *t* statistic, *P* value, and FDR, respectively, from the Treat method relative to fold-change threshold of 1.5. AveExpr, average log2 counts per million; FDR, false discovery rate; logFC, log2 fold-change; RNA-seq, RNA sequencing.
(XLSX)

S3 Data Differential accessibility analysis of ATAC-seq performed on Suz12 Wt and deleted (KO) basal-derived mammary organoids identified 2,767 windows by tiling 150 bp windows end to end across the genome. Columns A–D describe the location of the genomic window, E describes FDR, F the closest gene and its strand in G, H gene ID, I gene description, J Seqmonk element type, K window position relative to gene, L distance to gene, M–R, log2 RPKM for each experimental condition, S basal-derived organoid Wt average, T basal-derived KO average, and U the difference between the basal-derived KO and Wt. ATAC-seq, assay for transposase-accessible chromatin using sequencing; FDR, false discovery rate; KO, knockout; RPKM, reads per kilobase per million mapped reads; Suz12, Suppressor of Zeste 12 protein homolog; Wt, wild-type.
(XLSX)

S4 Data The 218 genes identified by both ATAC-seq and RNA-seq analysis as overrepresented in *Suz12*-deleted compared with Wt basal-derived mammary organoids. ATAC-seq, assay for transposase-accessible chromatin using sequencing; RNA-seq, RNA sequencing; Wt, wild-type.
(XLSX)

S5 Data List of the most significant GO Biological Process terms associated with the 218 overlapping gene list identified from ATAC-seq and RNA-seq analysis in *Suz12*-deleted compared with Wt basal-derived mammary organoids. ATAC-seq, assay for transposase-accessible chromatin using sequencing; GO, Gene ontology; RNA-seq, RNA sequencing; Wt, wild-type.
(XLSX)

S6 Data Individual quantitative observations that underlie the data summarized in the figures and results of this paper can be found here. (XLSX)

## Abbreviations

4OHT: 4-hydroxytamoxifen
ATAC-seq: assay for transposase-accessible chromatin using sequencing
CC3: cleaved caspase 3
cpm: counts per million
DA: differentially accessible
DE: differentially expressed
EED: embryonic ectoderm development
ER: estrogen receptor
Ezh2: Enhancer of Zeste homolog 2
Foxa1: forkhead box A1
GO: Gene ontology
H3K27me1: histone 3 lysine 27 monomethylation
H3K27me2: histone 3 lysine 27 dimethylation
H3K27me3: histone 3 lysine 27 trimethylation
HE: hematoxylin–eosin
IF: immunofluorescence
IHC: immunohistochemistry
MACS: model-based analysis of ChIP-seq
MaSC: mammary stem cell
MD: mean-difference
MEC: mammary epithelial cell
MMTVcre: cre transgenic mice that express Cre under control of the mouse mammary tumor virus promoter
p19Arf: p19 alternate reading frame
PARP: poly ADP ribose polymerase
PR: progesterone receptor
PRC2: Polycomb repressive complex 2
PTM: posttranslational modification
qRT-PCR: quantitative reverse-transcriptase PCR
R26creERT2: Rosa26-creERT2
RNA-seq: RNA sequencing
Suz12: suppressor of Zeste 12 protein homolog
TF: transcription factor
TMM: trimmed mean of M-values
TSS: transcriptional start site
Wt: wild-type

## References

[1] Ram O, Ram O, Goren A, Goren A, Amit I, Amit I, et al Combinatorial patterning of chromatin regulators uncovered by genome-wide location analysis in human cells. Cell. 2011;147:1628–39. 10.1016/j.cell.2011.09.057 22196736 PMC3312319

[2] Shen X, Shen X, Liu Y, Liu Y, Hsu Y-J, Hsu Y-J, et al EZH1 mediates methylation on histone H3 lysine 27 and complements EZH2 in maintaining stem cell identity and executing pluripotency. Mol. Cell. 2008;32:491–502. 10.1016/j.molcel.2008.10.016 19026780 PMC2630502

[3] Margueron R, Reinberg D. The Polycomb complex PRC2 and its mark in life. Nature. 2011;469:343–9. 10.1038/nature09784 21248841 PMC3760771

[4] Margueron R, Reinberg D. Chromatin structure and the inheritance of epigenetic information. Nat. Rev. Genet. 2010;11:285–96. 10.1038/nrg2752 20300089 PMC3760772

[5] Conway E, Healy E, Bracken AP. PRC2 mediated H3K27 methylations in cellular identity and cancer. Curr. Opin. Cell Biol. 2015;37:42–8. 10.1016/j.ceb.2015.10.003 26497635

[6] Aloia L, Di Stefano B, Di Croce L. Polycomb complexes in stem cells and embryonic development. Development. Oxford University Press for The Company of Biologists Limited; 2013;140:2525–34. 10.1242/dev.091553 23715546

[7] Boyer LA, Plath K, Zeitlinger J, Brambrink T, Medeiros LA, Lee TI, et al Polycomb complexes repress developmental regulators in murine embryonic stem cells. Nature. 2006;441:349–53. 10.1038/nature04733 16625203

[8] Chamberlain SJ, Yee D, Magnuson T. Polycomb repressive complex 2 is dispensable for maintenance of embryonic stem cell pluripotency. Stem Cells. John Wiley & Sons, Ltd; 2008;26:1496–505. 10.1634/stemcells.2008-0102 PMC2630378 18403752

[9] Pasini D, Bracken AP, Helin K. Polycomb group proteins in cell cycle progression and cancer. Cell Cycle. 2004;3:396–400. 14752272

[10] Ezhkova E, Lien W-H, Lien W-H, Stokes N, Pasolli HA, Silva JM, et al EZH1 and EZH2 cogovern histone H3K27 trimethylation and are essential for hair follicle homeostasis and wound repair. Genes Dev. Cold Spring Harbor Lab; 2011;25:485–98. 10.1101/gad.2019811 PMC3049289 21317239

[11] Bae WK, Kang K, Yu JH, Yoo KH, Factor VM, Kaji K, et al The methyltransferases enhancer of zeste homolog (EZH) 1 and EZH2 control hepatocyte homeostasis and regeneration. FASEB J. 2015;29:1653–62. 10.1096/fj.14-261537 25477280 PMC4415007

[12] Michalak EM, Visvader JE. Dysregulation of histone methyltransferases in breast cancer—Opportunities for new targeted therapies? Mol Oncol. 2016;10:1497–515. 10.1016/j.molonc.2016.09.003 27717710 PMC5423136

[13] Wassef M, Wassef M, Cragg MS, Rodilla V, Rodilla V, Phipson B, et al Impaired PRC2 activity promotes transcriptional instability and favors breast tumorigenesis. Genes Dev. Cold Spring Harbor Lab; 2015;29:2547–62. 10.1101/gad.269522.115 PMC4699384 26637281

[14] Li X, Li X, Gonzalez ME, Toy K, Filzen T, Filzen T, et al Targeted overexpression of EZH2 in the mammary gland disrupts ductal morphogenesis and causes epithelial hyperplasia. Am. J. Pathol. 2009;175:1246–54. 10.2353/ajpath.2009.090042 19661437 PMC2731143

[15] Visvader JE, Stingl J. Mammary stem cells and the differentiation hierarchy: current status and perspectives. Genes Dev. Cold Spring Harbor Lab; 2014;28:1143–58. 10.1101/gad.242511.114 PMC4052761 24888586

[16] Pal B, Bouras T, Shi W, Vaillant F, Sheridan JM, Fu N, et al Global changes in the mammary epigenome are induced by hormonal cues and coordinated by Ezh2. Cell Rep. 2013;3:411–26. 10.1016/j.celrep.2012.12.020 23375371

[17] Michalak EM, Nacerddine K, Pietersen A, Beuger V, Pawlitzky I, Cornelissen-Steijger P, et al Polycomb group gene Ezh2 regulates mammary gland morphogenesis and maintains the luminal progenitor pool. Stem Cells. 2013;31:1910–20. 10.1002/stem.1437 23712803

[18] Jamieson PR, Dekkers JF, Rios AC, Fu NY, Lindeman GJ, Visvader JE. Derivation of a robust mouse mammary organoid system for studying tissue dynamics. Development. 2016. 10.1242/dev.145045 27993977

[19] Wagner KU, McAllister K, Ward T, Davis B, Wiseman R, Hennighausen L. Spatial and temporal expression of the Cre gene under the control of the MMTV-LTR in different lines of transgenic mice. Transgenic Res. 2001;10:545–53. 11817542 10.1023/a:1013063514007

[20] Galvis LA, Galvis LA, Holik AZ, Holik AZ, Short KM, Short KM, et al Repression of Igf1 expression by Ezh2 prevents basal cell differentiation in the developing lung. Development. 2015;142:1458–69. 10.1242/dev.122077 25790853 PMC4392602

[21] Fu NY, Rios AC, Pal B, Soetanto R, Soetanto R, Lun ATL, et al EGF-mediated induction of Mcl-1 at the switch to lactation is essential for alveolar cell survival. Nat. Cell Biol. 2015;17:365–75. 10.1038/ncb3117 25730472

[22] McBryan J, Howlin J. Pubertal Mammary Gland Development: Elucidation of In Vivo Morphogenesis Using Murine Models Mammary Gland Development. New York, NY: Humana Press, New York, NY; 2017 pp. 77–114. 10.1007/978-1-4939-6475-8_3 27796948

[23] Xie H, Xu J, Hsu JH, Nguyen M, Fujiwara Y, Peng C, et al Polycomb repressive complex 2 regulates normal hematopoietic stem cell function in a developmental-stage-specific manner. Cell Stem Cell. 2014;14:68–80. 10.1016/j.stem.2013.10.001 24239285 PMC3947409

[24] Buenrostro JD, Giresi PG, Zaba LC, Chang HY, Greenleaf WJ. Transposition of native chromatin for fast and sensitive epigenomic profiling of open chromatin, DNA-binding proteins and nucleosome position. Nat. Methods. 2013;10:1213–8. 10.1038/nmeth.2688 24097267 PMC3959825

[25] Lim E, Vaillant F, Wu D, Forrest NC, Pal B, Hart AH, et al Aberrant luminal progenitors as the candidate target population for basal tumor development in BRCA1 mutation carriers. Nat. Med. 2009;15:907–13. 10.1038/nm.2000 19648928

[26] Cai S, Cai S, Kalisky T, Kalisky T, Sahoo D, Sahoo D, et al A Quiescent Bcl11b High Stem Cell Population Is Required for Maintenance of the Mammary Gland. Cell Stem Cell. 2017;20:247–260.e5. 10.1016/j.stem.2016.11.007 28041896 PMC5341693

[27] Yoo KH, Oh S, Kang K, Hensel T, Robinson GW, Hennighausen L. Loss of EZH2 results in precocious mammary gland development and activation of STAT5-dependent genes. Nucleic Acids Res. 2015;43:8774–89. 10.1093/nar/gkv776 26250110 PMC4605299

[28] Pellacani D, Bilenky M, Kannan N, Heravi-Moussavi A, Knapp DJHF, Gakkhar S, et al Analysis of Normal Human Mammary Epigenomes Reveals Cell-Specific Active Enhancer States and Associated Transcription Factor Networks. Cell Rep. 2016;17:2060–74. 10.1016/j.celrep.2016.10.058 27851968

[29] Fu NY, Rios AC, Pal B, Law CW, Jamieson P, Liu R, et al Identification of quiescent and spatially restricted mammary stem cells that are hormone responsive. Nat. Cell Biol. Nature Publishing Group; 2017;19:164–76. 10.1038/ncb3471 28192422

[30] Bae WK, Hennighausen L. Canonical and non-canonical roles of the histone methyltransferase EZH2 in mammary development and cancer. Mol. Cell. Endocrinol. 2014;382:593–7. 10.1016/j.mce.2013.05.002 23684884 PMC3843995

[31] Bae WK, Yoo KH, Lee JS, Kim Y, Chung I-J, Park MH, et al The methyltransferase EZH2 is not required for mammary cancer development, although high EZH2 and low H3K27me3 correlate with poor prognosis of ER-positive breast cancers. Mol. Carcinog. 2014;54:1172–80. 10.1002/mc.22188 25043748 PMC4286524

[32] Bracken AP, Kleine-Kohlbrecher D, Kleine-Kohlbrecher D, Dietrich N, Dietrich N, Pasini D, et al The Polycomb group proteins bind throughout the INK4A-ARF locus and are disassociated in senescent cells. Genes Dev. Cold Spring Harbor Lab; 2007;21:525–30. 10.1101/gad.415507 PMC1820894 17344414

[33] Rivandi M, Khorrami M-S, Fiuji H, Shahidsales S, Hasanzadeh M, Jazayeri MH, et al The 9p21 locus: A potential therapeutic target and prognostic marker in breast cancer. J. Cell. Physiol. Wiley-Blackwell; 2018;233:5170–9. 10.1002/jcp.26332 29240242

[34] Lee SCW, Lee SCW, Miller S, Miller S, Hyland C, Hyland C, et al Polycomb repressive complex 2 component Suz12 is required for hematopoietic stem cell function and lymphopoiesis. Blood. 2015;126:167–75. 10.1182/blood-2014-12-615898 26036803

[35] Neff T, Neff T, Sinha AU, Sinha AU, Kluk MJ, Kluk MJ, et al Polycomb repressive complex 2 is required for MLL-AF9 leukemia. Proc. Natl. Acad. Sci. U.S.A. 2012;109:5028–33. 10.1073/pnas.1202258109 22396593 PMC3324004

[36] Su I-H, Basavaraj A, Basavaraj A, Krutchinsky AN, Krutchinsky AN, Hobert O, et al Ezh2 controls B cell development through histone H3 methylation and Igh rearrangement. Nat. Immunol. 2003;4:124–31. 10.1038/ni876 12496962

[37] Wagner KU, Erlacher M, Wall RJ, Labi V, St-Onge L, Manzl C, et al Cre-mediated gene deletion in the mammary gland. Nucleic Acids Res. Oxford University Press; 1997;25:4323–30. 10.1093/nar/25.21.4323 PMC147032 9336464

[38] Seibler J, Zevnik B, Küter-Luks B, Andreas S, Kern H, Hennek T, et al Rapid generation of inducible mouse mutants. Nucleic Acids Res. 2003;31:e12 12582257 10.1093/nar/gng012 PMC150244

[39] Vasanthakumar A, Xu D, Lun AT, Kueh AJ, van Gisbergen KP, Iannarella N, et al A non-canonical function of Ezh2 preserves immune homeostasis. EMBO Rep. 2017;18:619–31. 10.15252/embr.201643237 28223321 PMC5376973

[40] Shackleton M, Vaillant F, Simpson KJ, Simpson KJ, Stingl J, Smyth GK, et al Generation of a functional mammary gland from a single stem cell. Nature. 2006;439:84–8. 10.1038/nature04372 16397499

[41] Langmead B, Salzberg SL. Fast gapped-read alignment with Bowtie 2. Nat. Methods. Nature Publishing Group, a division of Macmillan Publishers Limited. All Rights Reserved; 2012;9:357–9. 10.1038/nmeth.1923 PMC3322381 22388286

[42] Robinson MD, McCarthy DJ, Smyth GK. edgeR: a Bioconductor package for differential expression analysis of digital gene expression data. Bioinformatics. 2010;26:139–40. 10.1093/bioinformatics/btp616 19910308 PMC2796818

[43] Ramírez F, Ryan DP, Grüning B, Bhardwaj V, Kilpert F, Richter AS, et al deepTools2: a next generation web server for deep-sequencing data analysis. Nucleic Acids Res. Oxford University Press; 2016;44:W160–5. 10.1093/nar/gkw257 PMC4987876 27079975

[44] Liao Y, Smyth GK, Shi W. The Subread aligner: fast, accurate and scalable read mapping by seed-and-vote. Nucleic Acids Res. 2013;41:e108 10.1093/nar/gkt214 23558742 PMC3664803

[45] Liao Y, Smyth GK, Shi W. featureCounts: an efficient general purpose program for assigning sequence reads to genomic features. Bioinformatics. 2014;30:923–30. 10.1093/bioinformatics/btt656 24227677

[46] Ritchie ME, Phipson B, Wu D, Hu Y, Law CW, Shi W, et al limma powers differential expression analyses for RNA-sequencing and microarray studies. Nucleic Acids Res. 2015;43:e47 10.1093/nar/gkv007 25605792 PMC4402510

[47] Robinson MD, Oshlack A. A scaling normalization method for differential expression analysis of RNA-seq data. Genome Biol. BioMed Central; 2010;11:R25. 10.1186/gb-2010-11-3-r25 PMC2864565 20196867

[48] Smyth GK, Michaud J, Scott HS. Use of within-array replicate spots for assessing differential expression in microarray experiments. Bioinformatics. 2005;21:2067–75. 10.1093/bioinformatics/bti270 15657102

[49] McCarthy DJ, Smyth GK. Testing significance relative to a fold-change threshold is a TREAT. Bioinformatics. 2009;25:765–71. 10.1093/bioinformatics/btp053 19176553 PMC2654802

[50] Phipson B, Lee S, Majewski IJ, Alexander WS, Smyth GK. ROBUST HYPERPARAMETER ESTIMATION PROTECTS AGAINST HYPERVARIABLE GENES AND IMPROVES POWER TO DETECT DIFFERENTIAL EXPRESSION. Ann Appl Stat. 2016;10:946–63. 10.1214/16-AOAS920 28367255 PMC5373812

[51] Prat A, Parker JS, Karginova O, Fan C, Livasy C, Livasy C, et al Phenotypic and molecular characterization of the claudin-low intrinsic subtype of breast cancer. Breast Cancer Res. 2010;12:R68 10.1186/bcr2635 20813035 PMC3096954

[52] Silver JD, Ritchie ME, Smyth GK. Microarray background correction: maximum likelihood estimation for the normal-exponential convolution. Biostatistics. 2009;10:352–63. 10.1093/biostatistics/kxn042 19068485 PMC2648902

