# Supplementary figures and images for: Canonical PRC2 function is essential for mammary gland development and affects chromatin compaction in mammary organoids
Source: PLoS Biol. 2018 Aug 6;16(8):e2004986. doi: 10.1371/journal.pbio.2004986 (PMC6095611; doi:10.1371/journal.pbio.2004986)

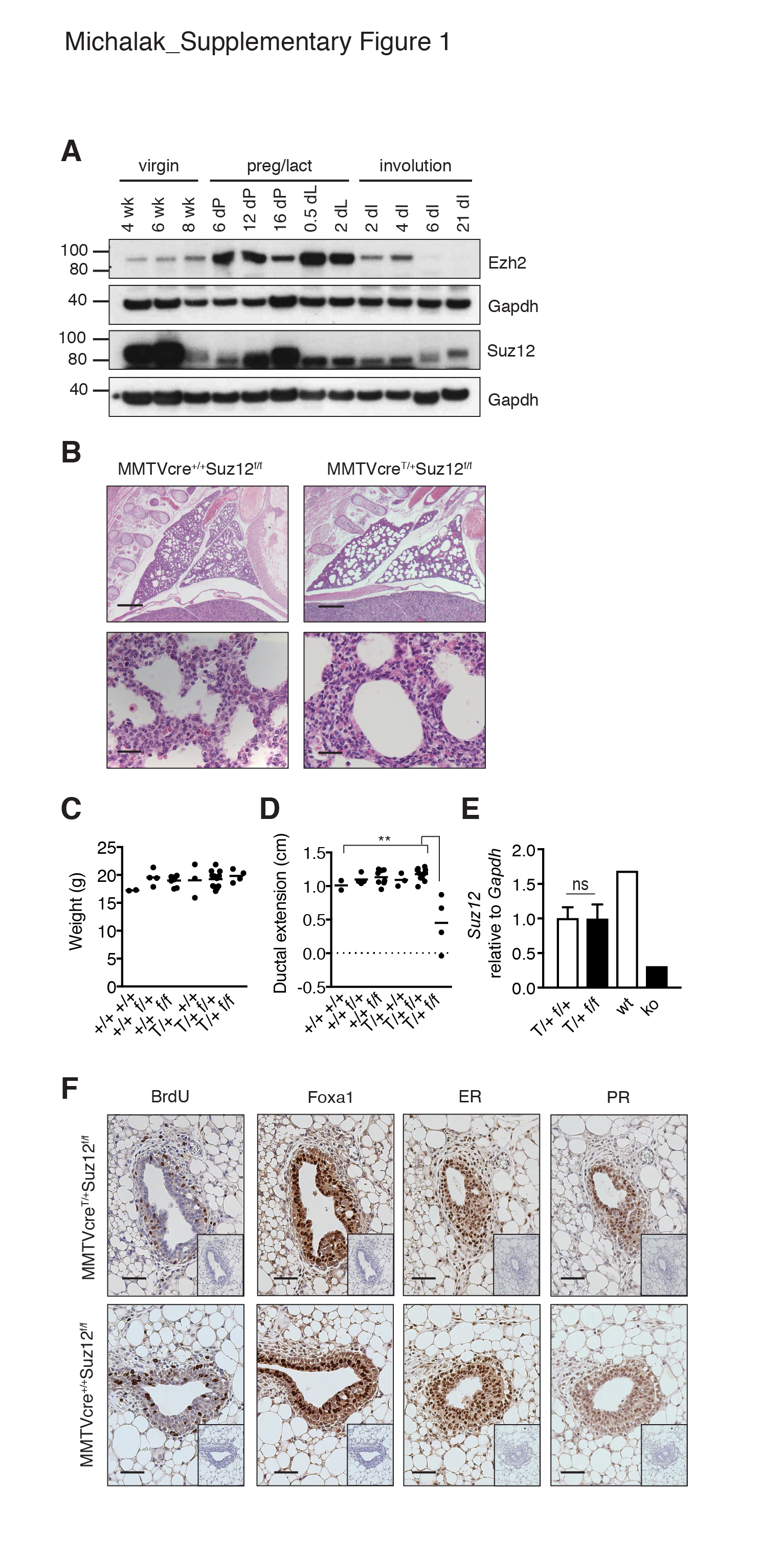

Supplement: S1 Fig — (A) Western blot for Ezh2 and Suz12 in whole mammary gland lysates prepared at the indicated developmental timepoints. Gapdh serves as a loading control. Molecular mass in KDa of the protein ladder is shown on the left-hand side. (B) Representative images of HE-stained sections of lungs from MMTVcreT/+Suz12f/f and control littermates recovered by caesarian section at E19.5. Examination of HE-stained sections revealed that while lungs appear to be normal in size (top), at higher magnification (bottom), lungs from MMTVcreT/+Suz12f/f mice have fewer septa and increased mesenchyme, consistent with abnormal lung differentiation. Scale bars: 1.5 mm (top) and 50 μm (bottom). (C) Weight of 5–6 week old MMTVcreT/+Suz12f/f mice and control littermates of the indicated genotypes. (D) Ductal extension of mammary glands from 5–6 week old MMTVcreT/+Suz12f/f mice and control littermates of the indicated genotypes. Ductal extension is calculated as the distance from the center of the lymph node to the leading edge of the mammary outgrowth in R4 mammary gland whole mounts. Individual data points and the mean are shown. ** P < 0.01 for T/+ f/f compared with all other genotypes (one-way ANOVA for multiple comparisons). (D) qRT-PCR analysis of Suz12 mRNA expression in mammary glands from 6 week old MMTVcreT/+Suz12f/+ and MMTVcreT/+Suz12f/f mice. Expression was calculated relative to Gapdh, and values were normalized to the average of the conditions per experiment. Mean ± S.E.M. (n = 4). R26creERT2KI/+Suz12f/f MECs treated without (Wt) or with 4OHT (ko) to delete Suz12 were used as controls (n = 1). One of two experiments with two independent sets of primer pairs for Suz12 is shown. (E) Representative images of immunohistochemical staining of terminal end buds in mammary glands from 6 week-old MMTVcreT/+Suz12f/f and control littermates. Markers of proliferation (BrdU) and differentiation of MECs into hormone receptor positive mammary subsets (Foxa1, ER, PR) were included. Isotype-contro [file pbio.2004986.s001.tif]

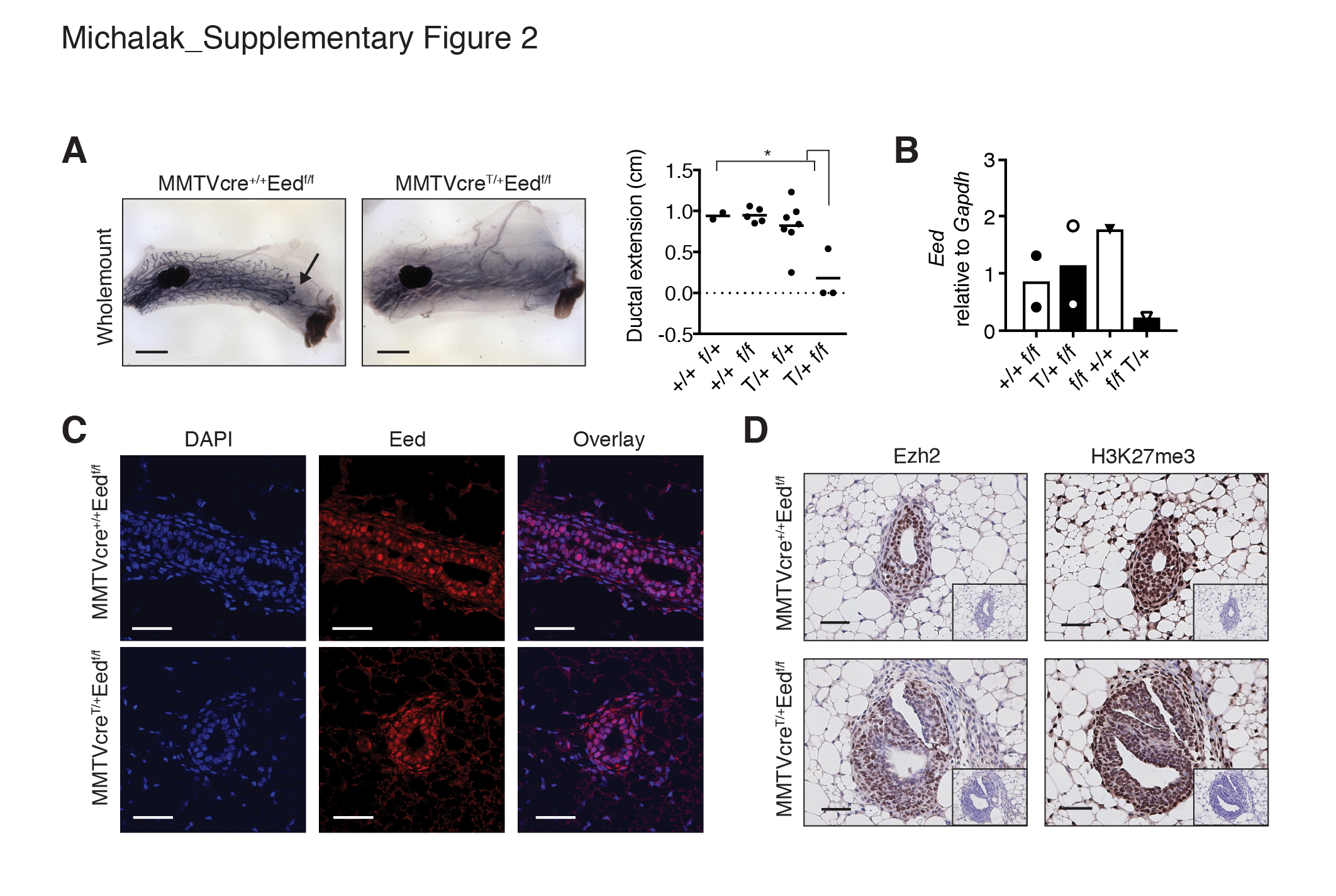

Supplement: S2 Fig — (A) Representative images of whole mounts (left) and ductal extension (right) of mammary glands from MMTVcreT/+Eedf/f mice and control littermates of the indicated genotypes. Arrows indicate the leading edge of the mammary epithelium. Scale bars: 4 mm. Ductal extension was calculated as described in S1 Fig. Individual data points and the mean are shown. * P < 0.05 for T/+ f/f compared with all other genotypes (one-way ANOVA for multiple comparisons). (B) qRT-PCR analysis of Eed mRNA expression in mammary glands from 6–7 week old MMTVcre+/+Eedf/f and MMTVcreT/+Eedf/f mice. Expression was calculated relative to Gapdh, and values were normalized to the average of the conditions per experiment. Mean and individual values are shown (n = 2). CD4cre+/+Eedf/f (f/f +/+) and CD4creT/+Eedf/f (f/f T/+) T lymphocytes were used as controls (n = 1). One of two experiments with two independent sets of primer pairs for Eed is shown. (C) Immunofluorescent staining for Eed in mammary glands from 6 week old MMTVcreT/+Eedf/f and control littermates. Scale bars: 50 μm. (D) Immunohistochemical staining for Ezh2 and H3K27me3 in mammary glands from 6 week old MMTVcreT/+Eedf/f and control littermates. Isotype-control stained sections are shown in the inset. Scale bars: 50 μm. Individual quantitative observations can be found in S6 Data. Eed, embryonic ectoderm development; H3K27me3; histone 3 lysine 27 trimethylation; qRT-PCR, quantitative reverse-transcriptase PCR. (TIF) [file pbio.2004986.s002.tif]

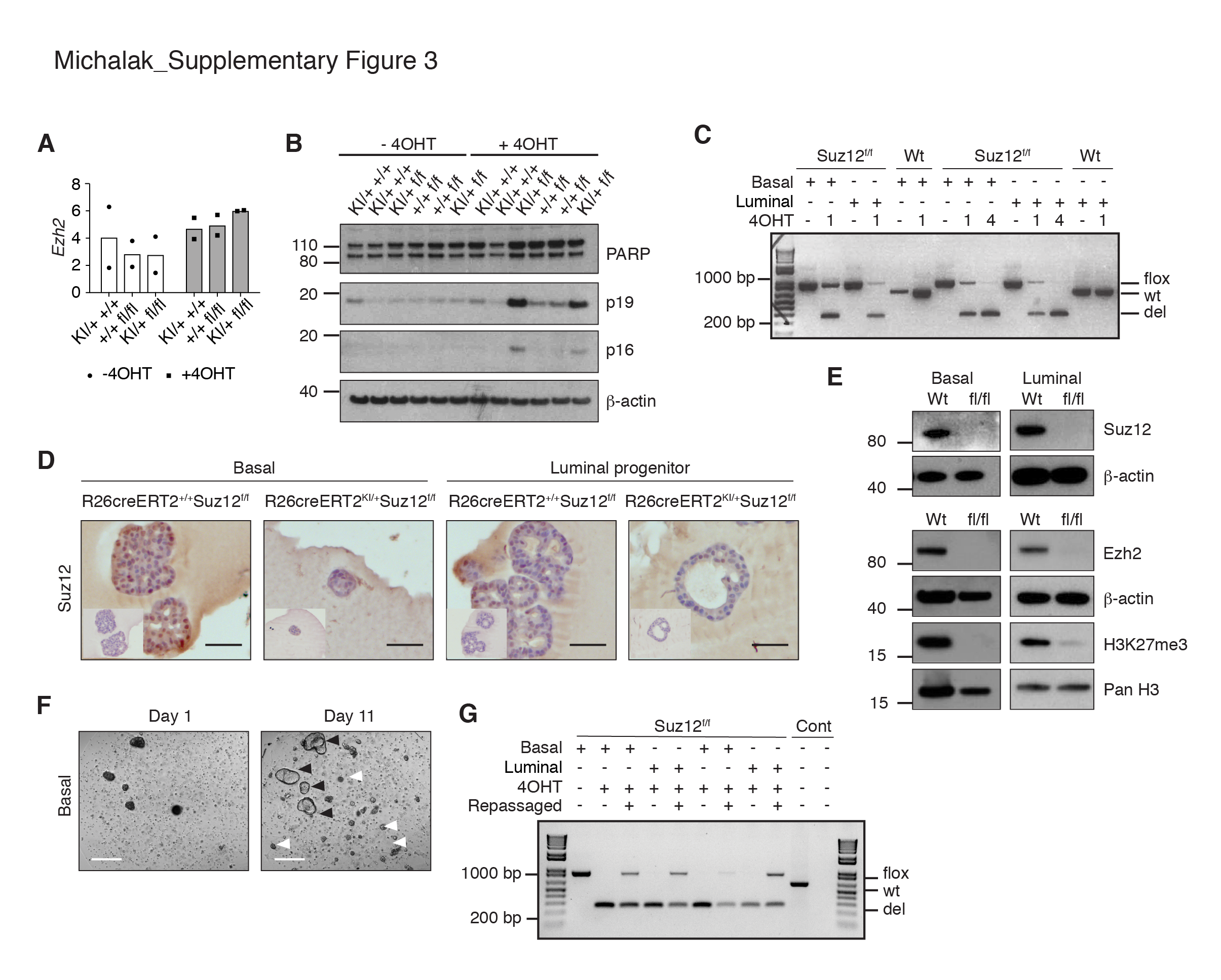

Supplement: S3 Fig — (A) qRT-PCR analysis of Ezh2 mRNA expression in MEC from R26creERT2KI/+Suz12f/f mice and the indicated control genotypes following addition of 4OHT to induce Suz12 deletion on day 2. Cells were cultured for 1 week prior to preparation of RNA. Copies of Ezh2 are expressed relative to GAPDH. (B) Western blot analysis of protein expression in MECs from R26creERT2KI/+Suz12f/f mice and the indicated control genotypes following addition of 4OHT to induce Suz12 deletion on day 2. Cells were cultured for 1 week prior to preparation of protein lysates. Molecular mass in KDa of the protein ladder are shown on the left. (C) Image of genotyping PCR performed on organoids grown for 2 weeks from single basal or luminal progenitor cells from R26creERT2KI/+Suz12f/f mice or Wt mice. Organoids were left untreated (-) or treated with 4OHT on day 1 (1) or day 4 (4) of culture. The size of Suz12 Wt, floxed (flox), and recombined (del) alleles are indicated. The size (bp) of the DNA ladder is shown on the left-hand side. (D) Immunohistochemical staining for Suz12 on 2 week old organoids from R26creERT2KI/+Suz12f/f or control mice, treated with 4OHT on day 4 of culture. Control stained sections are shown in the inset. Scale bars: 400 μm. (E) Western blot analysis of 2 week old organoids from R26creERT2KI/+Suz12f/f mice or control mice, treated with 4OHT on day 4 of culture. Molecular mass in KDa of the protein ladder is shown on the left-hand side. (F) Representative images of repassaged organoids grown for 2 weeks from single basal cells from R26creERT2KI/+Suz12f/f mice, on day 1 and day 11 after passaging. Black arrowheads indicate clumps of cells that became cystic overnight after passaging. White arrowheads represent new noncystic colonies that formed from single cells. Scale bars: 200 μm. (G) Image of genotyping PCR performed on primary or repassaged organoids described in (B) after 11 days in culture. The size of Suz12 Wt, flox, and del alleles are indicated. Basal- or luminal-deriv [file pbio.2004986.s003.tif]

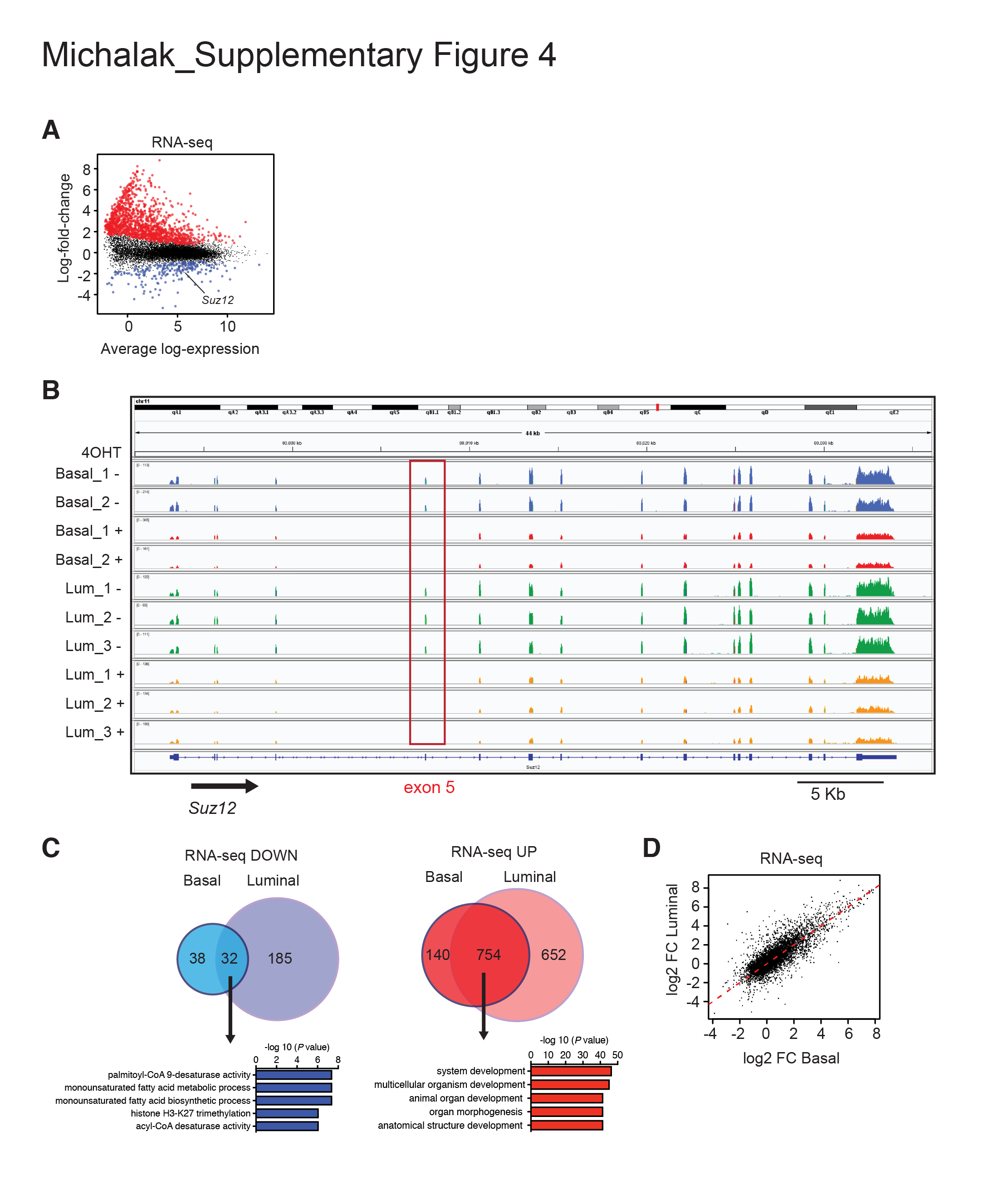

Supplement: S4 Fig — (A) MD plot showing log2-fold expression changes versus average log2-expression by RNA-sequencing in luminal-derived organoids deleted for Suz12. Up- and down-regulated genes with changes significantly greater than 1.5-fold are highlighted in red and blue, respectively (Treat-FDR < 0.05). Suz12 expression is indicated. (B) IGV profile of RNA-sequencing reads for Suz12 in basal- or luminal-derived organoids left untreated or treated with 4OHT on day 4 of culture. The metrics are shown on the left side of each plot for each sample. The direction of transcription is marked by an arrow. Exon 5, around which loxP sites are situated in the Suz12 floxed allele, is indicated by the boxed region. Scale bar: 5 Kb. (C) Venn diagrams showing the number of DE down-regulated (left) and up-regulated (right) genes in Suz12-deleted basal- and luminal-derived organoids. Overlap in DE genes is indicated, as well as the 5 top GO terms for that gene set. (D) Scatterplot relating log2-fold gene expression changes in Suz12-deleted basal- and luminal-derived organoids. Changes in basal-derived (x-axis) and luminal-derived (y-axis) organoids are highly correlated (P < 1e-16). Individual quantitative observations can be found in S6 Data. 4OHT, 4-hydroxytamoxifen; DE, differentially expressed; FDR, false discovery rate; GO, Gene ontology; IGV, Integrative Genomics Viewer; MD, mean-difference; Suz12, Suppressor of Zeste 12 protein homolog. (TIF) [file pbio.2004986.s004.tif]

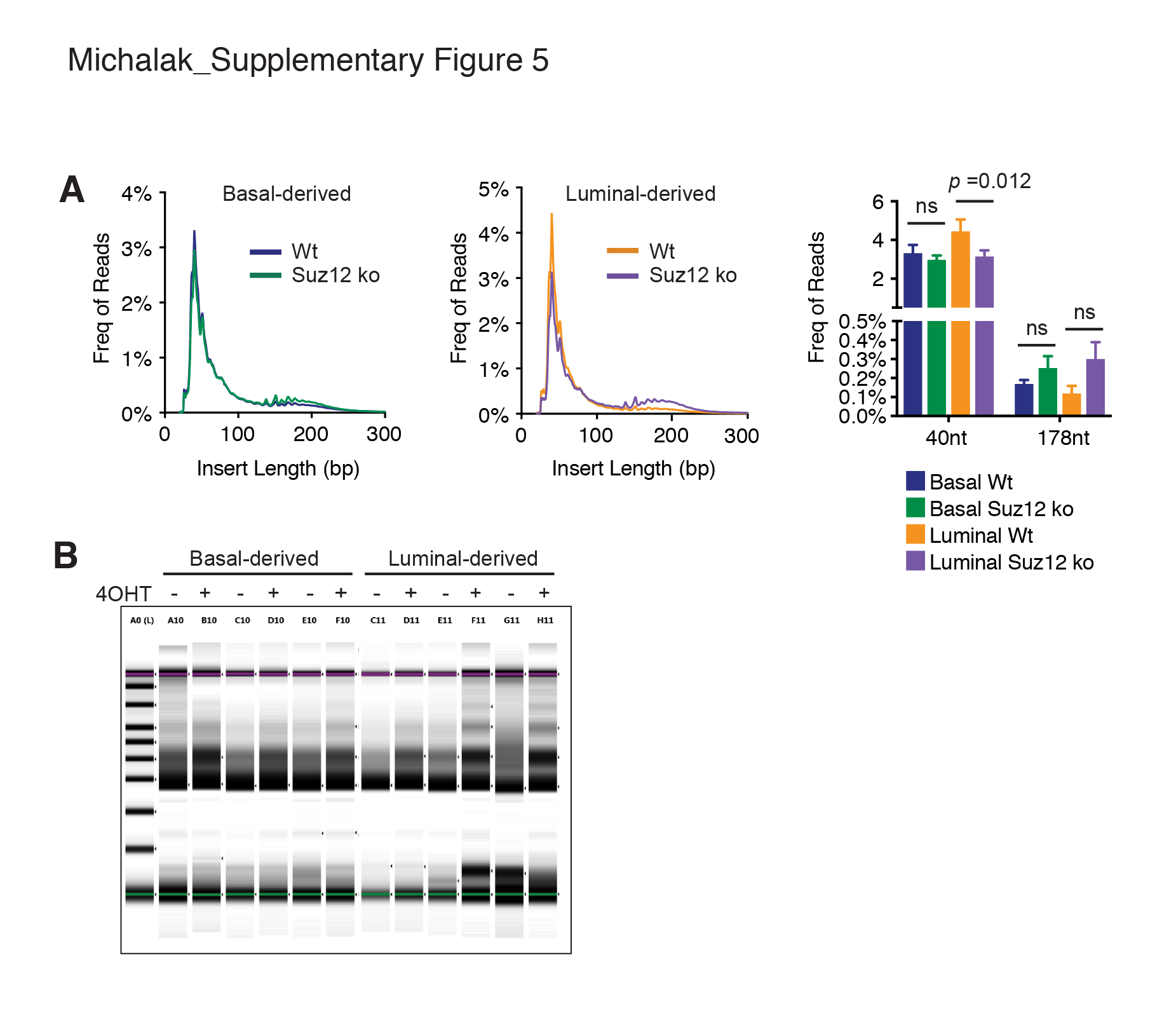

Supplement: S5 Fig — (A) The averaged frequency of read insert length obtained by ATAC-seq for Wt and Suz12-deleted (ko) basal- and luminal-derived organoids relative to distance from the TSS is shown. The 40 and 178 nt peaks correspond to nucleosome-free regions and the mononucleosome, respectively. (B) An image of the ATAC-seq libraries for Wt (-) or Suz12-deleted (+) basal- and luminal-derived organoids, resolved on Tape-station. The ladder is in the first lane on the left-hand side. Individual quantitative observations can be found in S6 Data. ATAC-seq, assay for transposase-accessible chromatin using sequencing; ko, knockout; nt, nucleotide; TSS, transcriptional start site; Wt, wild-type. (TIF) [file pbio.2004986.s005.tif]

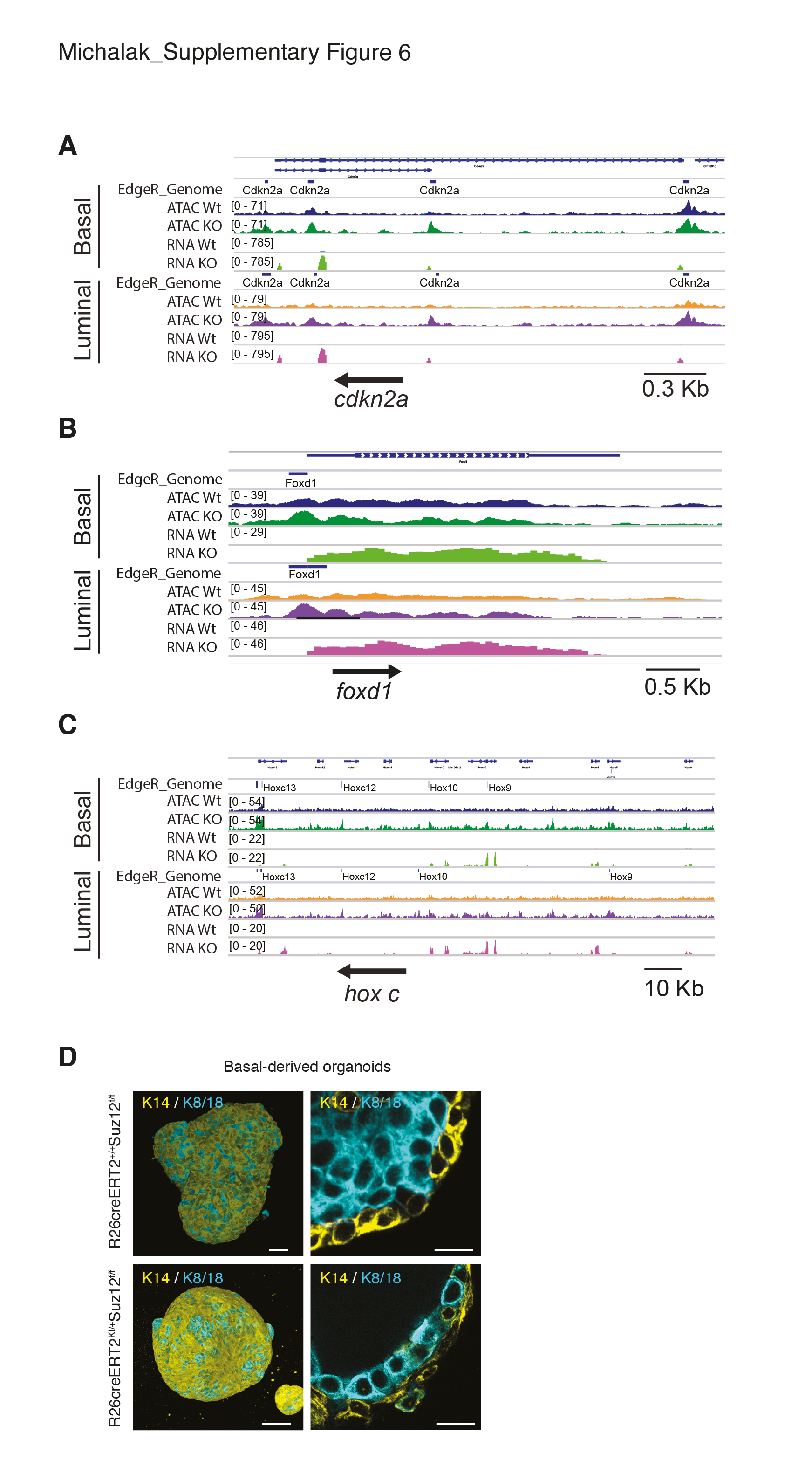

Supplement: S6 Fig — The RNA-seq and ATAC-seq profiles around Cdkn2a (A), Foxd1 (B), and Hoxc locus (C) are shown. The metrics are shown on the left side of each plot for each sample. The direction of transcription is marked by an arrow. Scale bars are indicated. (D) A whole-mount 3D confocal image (left) and an optical section (right) of a Wt and Suz12-deleted (KO) basal-derived organoid labeled for K14 and K8/18. Scale bars: 30 μm (whole mount) and 15 μm (section). ATAC-seq, assay for transposase-accessible chromatin using sequencing; RNA-seq, RNA sequencing; Wt, wild-type. (TIF) [file pbio.2004986.s006.tif]

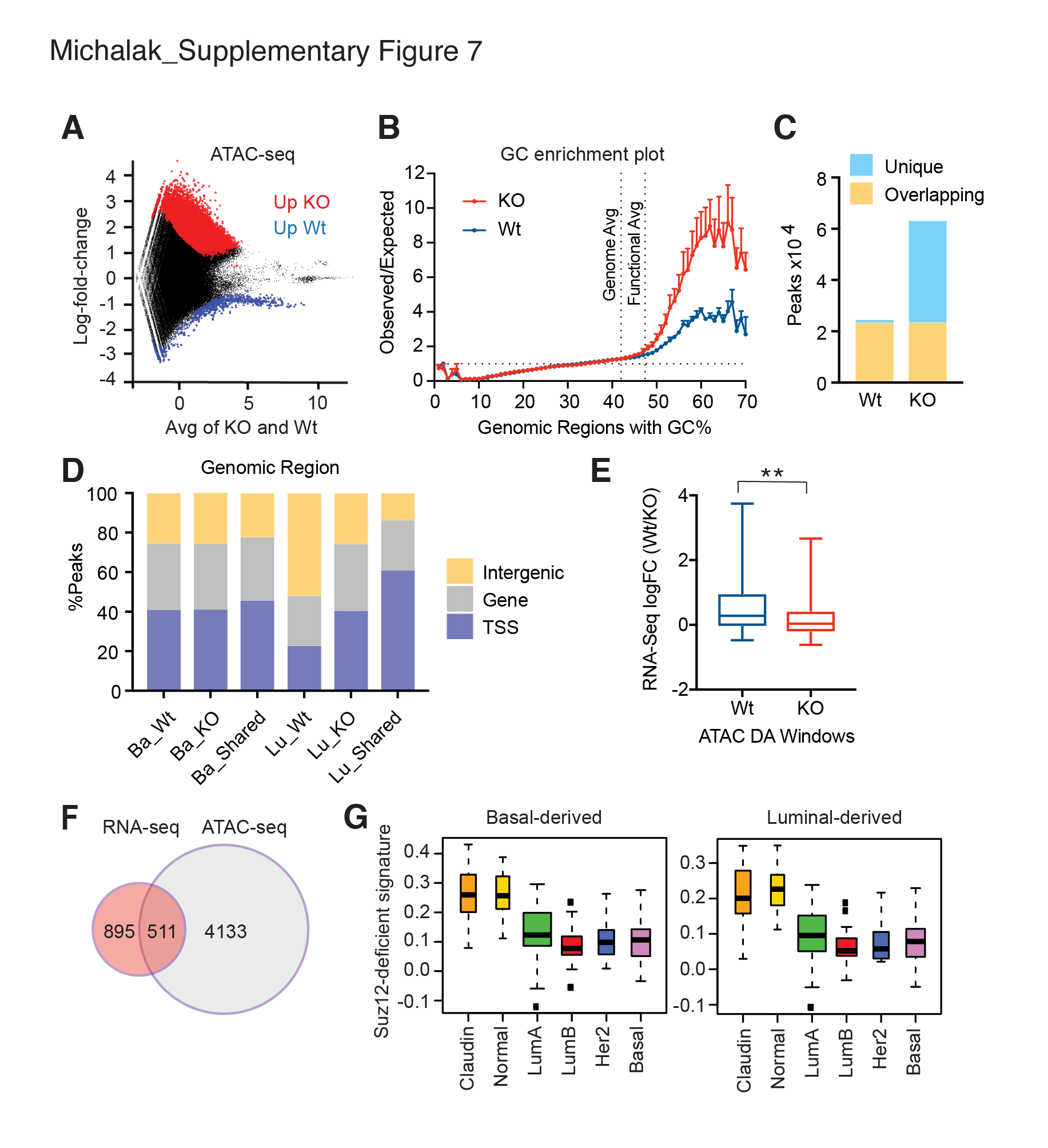

Supplement: S7 Fig — (A) MD plot showing 150 bp windows identified as significantly DA (log-fold change) by ATAC-seq analysis of Suz12-deleted (KO) luminal-derived organoids deleted for Suz12, compared with Wt average log-expression. Regions that were significantly up- or down-regulated are shown in red or blue, respectively. (B) A plot showing GC nucleotide enrichment in genomic regions of ATAC-Seq reads of Suz12-deleted (KO) luminal-derived organoids, compared with Wt. Shown are the genomic and functional DNA element average for the mouse genome. (C) MACS Peaks calling analysis of Suz12-deleted (KO) luminal-derived organoids, compared with Wt. (D) Analysis of genomic regions associated with significantly DA windows identified by ATAC-seq analysis of Wt and Suz12-deleted (KO) basal- or luminal-derived organoids. (E) Box and whisker plot of significantly DA genes found to be associated with windows found in (A) by ATAC-seq analysis of Wt and Suz12-deleted (KO) luminal-derived organoids, and their corresponding expression by RNA-seq analysis. Whiskers represent the 5%–95% intervals. ** P < 0.0028 (unpaired t test, Welch’s correction) (F) Venn diagram showing the overlap in up-regulated DE genes between RNA-seq and DA genes by ATAC-seq in Suz12-deleted luminal-derived organoids. (G) Suz12-deleted transcriptional signature by breast cancer tumor subtype (Claudin-low, Normal-like, Luminal A, Luminal B, Her2-positive, Basal-like). Box plots show the aggregate gene expression score in each tumor subtype [51] for genes associated with Suz12-deficiency in basal-derived organoids (left) and luminal-derived organoids (right). The Suz12-deficient expression score is highest in the claudin-low subtype and lowest in the basal and Her2 subtypes (P = 3.8e-10 for basal-derived and i = 6.2e-11 for luminal-derived organoids by Tukey HSD). ATAC-seq, assay for transposase-accessible chromatin using sequencing; DA, differentially accessible; DE, differentially expressed; Her2, human epidermal growth factor [file pbio.2004986.s007.tif]
